# An *Irak1-Mecp2* tandem duplication mouse model for the study of *MECP2* duplication syndrome

**DOI:** 10.1242/dmm.050528

**Published:** 2024-07-23

**Authors:** Eleonora Maino, Ori Scott, Samar Z. Rizvi, Wing Suen Chan, Shagana Visuvanathan, Youssif Ben Zablah, Hongbin Li, Ameet S. Sengar, Michael W. Salter, Zhengping Jia, Janet Rossant, Ronald D. Cohn, Bin Gu, Evgueni A. Ivakine

**Affiliations:** ^1^ Program in Genetics and Genome Biology, the Hospital for Sick Children, Toronto, ON M5G 0A4, Canada; ^2^ Department of Molecular Genetics, University of Toronto, Toronto, ON M5S 1A8, Canada; ^3^ Division of Clinical Immunology and Allergy, Department of Pediatrics, the Hospital for Sick Children and University of Toronto, Toronto, ON M5G 1E8, Canada; ^4^ Department of Physiology, University of Toronto, Toronto, ON M5S 1A8, Canada; ^5^ Program in Neuroscience and Mental Health, the Hospital for Sick Children, Toronto, ON M5G 0A4, Canada; ^6^ Program in Developmental and Stem Cell Biology, the Hospital for Sick Children Research Institute, Toronto, ON M5G 0A4, Canada; ^7^ Division of Clinical and Metabolic Genetics, Department of Pediatrics, the Hospital for Sick Children and University of Toronto, Toronto, ON M5G 1X8, Canada; ^8^ Department of Obstetrics, Gynecology and Reproductive Biology, Michigan State University, East Lansing, MI 48824, USA; ^9^ Institute for Quantitative Health Science and Engineering, Michigan State University, East Lansing, MI 48824, USA

**Keywords:** *MECP2* duplication syndrome, IRAK1, Disease modeling, Intellectual disability, Interferon, Mouse model

## Abstract

*MECP2* duplication syndrome (MDS) is a neurodevelopmental disorder caused by tandem duplication of the *MECP2* locus and its surrounding genes, including *IRAK1*. Current MDS mouse models involve transgenic expression of *MECP2* only, limiting their applicability to the study of the disease. Herein, we show that an efficient and precise CRISPR/Cas9 fusion proximity-based approach can be utilized to generate an *Irak1-Mecp2* tandem duplication mouse model (‘*Mecp2 Dup*’). The *Mecp2 Dup* mouse model recapitulates the genomic landscape of human MDS by harboring a 160 kb tandem duplication encompassing *Mecp2* and *Irak1*, representing the minimal disease-causing duplication, and the neighboring genes *Opn1mw* and *Tex28*. The *Mecp2 Dup* model exhibits neuro-behavioral abnormalities, and an abnormal immune response to infection not previously observed in other mouse models, possibly owing to *Irak1* overexpression. The *Mecp2 Dup* model thus provides a tool to investigate MDS disease mechanisms and develop potential therapies applicable to patients.

## Teaser

**Summary:** A CRISPR/Cas9-based mouse model of *MECP2* duplication syndrome mirrors *IRAK1*-*MECP2* tandem duplications in patients, shedding light on neurobehavioral and immune complexities for therapeutic exploration.

## INTRODUCTION

MECP2 is a transcriptional regulator with a multi-faceted role in the development and function of various tissues, most notably within the central nervous system (D'Mello, 2021). Variants in *MECP2* underlie two pediatric neurodevelopmental disorders: Rett syndrome (RTT), caused by *MECP2* loss-of-function variants (Amir et al., 1999), and *MECP2* duplication syndrome (MDS), resulting from duplications of the X chromosome region Xq28 (Van Esch et al., 2005). These two neurodevelopmental disorders caused by opposite genetic alterations highlight the need for a precise MECP2 dosage within the nervous system. RTT affects one in 10,000 females and manifests as developmental regression, breathing abnormalities, seizures, anxiety and metabolic dysfunction (Kyle et al., 2018). MDS affects mainly boys, with an estimated live birth prevalence of one in 150,000 (Giudice-Nairn et al., 2019), and is suspected to underlie 1% of undiagnosed X-linked intellectual disabilities (Lugtenberg et al., 2009). It is characterized by developmental and psychomotor delay, locomotor and speech complications, autism spectrum disorder (ASD), seizures and susceptibility to infections (Ta et al., 2022). Life expectancy is drastically reduced, with patients often succumbing to respiratory tract infections (Friez et al., 2006).

From a genomic perspective, although RTT is primarily caused by point mutations affecting a single gene, MDS presents additional complexity as it can be caused by duplications of different sizes ranging from 50 kb to 15 Mb, affecting at least two genes: *IRAK1* and *MECP2* (Peters et al., 2019). As a result, faithful modeling of MDS remains a challenge. To date, MDS has primarily been investigated using transgenic mice overexpressing the murine or human *MECP2* transgene, such as the *Tau-Mecp2* (Koerner et al., 2018; Luikenhuis et al., 2004), *MECP2-TG* (Collins et al., 2004), *MECP2^R11G^-TG* and *MECP2^R306^-TG* (Heckman et al., 2014), and *hDup* (Shao et al., 2021a) mouse models. These models have been instrumental in elucidating certain pathophysiological mechanisms underlying the disease and demonstrating its reversibility (Sztainberg et al., 2015). However, none of these models recapitulate the genomic rearrangements harbored by most patients, caused by head-to-tail tandem duplication mutations. More importantly, they do not account for the duplication of *IRAK1*, which is shared by all patients with MDS and which may influence disease manifestations through its critical role in host immunity (Gottipati et al., 2008).

The need to model genomic copy number variations (CNVs) is not unique to MDS, as deletions and duplications of genomic regions underlie many human genetic disorders (Lupski, 2009). In particular, multigenic tandem duplications are associated with several disorders causing intellectual disability, ASD and developmental delay (Coe et al., 2019; Takumi and Tamada, 2018; Zarrei et al., 2019). With the advent of new CRISPR/Cas9 genome-editing technologies, mouse models recapitulating disease-causing CNVs have been generated, with efficient current approaches for generating deletions. However, generating model organisms recapitulating tandem duplications is still an inefficient, inaccurate and time-consuming process, leading to few such models to date (Maino et al., 2021; Pristyazhnyuk et al., 2019).

Here, we optimized the tandem duplication generation approach by utilizing a Cas9 fusion proximity-based strategy. We used this methodology to generate the *Mecp2 Dup* mouse model, harboring a 160 kb tandem duplication on the X chromosome. This duplication region encompasses both the *Irak1* and *Mecp2* loci, the minimal duplicated genes shared by patients with MDS*.* The *Mecp2 Dup* mouse model recapitulates neurobehavioral aspects of MDS and has an abnormal immune response to infection not previously described in the *Mecp2* overexpression models, possibly due to the impact of *Irak1* duplication. The *Mecp2 Dup* mouse model may serve as a platform to investigate disease mechanisms and develop novel therapeutic approaches for patients with MDS. Furthermore, the Cas9 fusion proximity-based approach can be used more broadly as an efficient and precise method of generating models of complex genomic rearrangements.

## RESULTS

### A Cas9 fusion proximity-based approach generates the *Mecp2 Dup* and *Mecp2 Del* mouse models

**Fig. 1.**
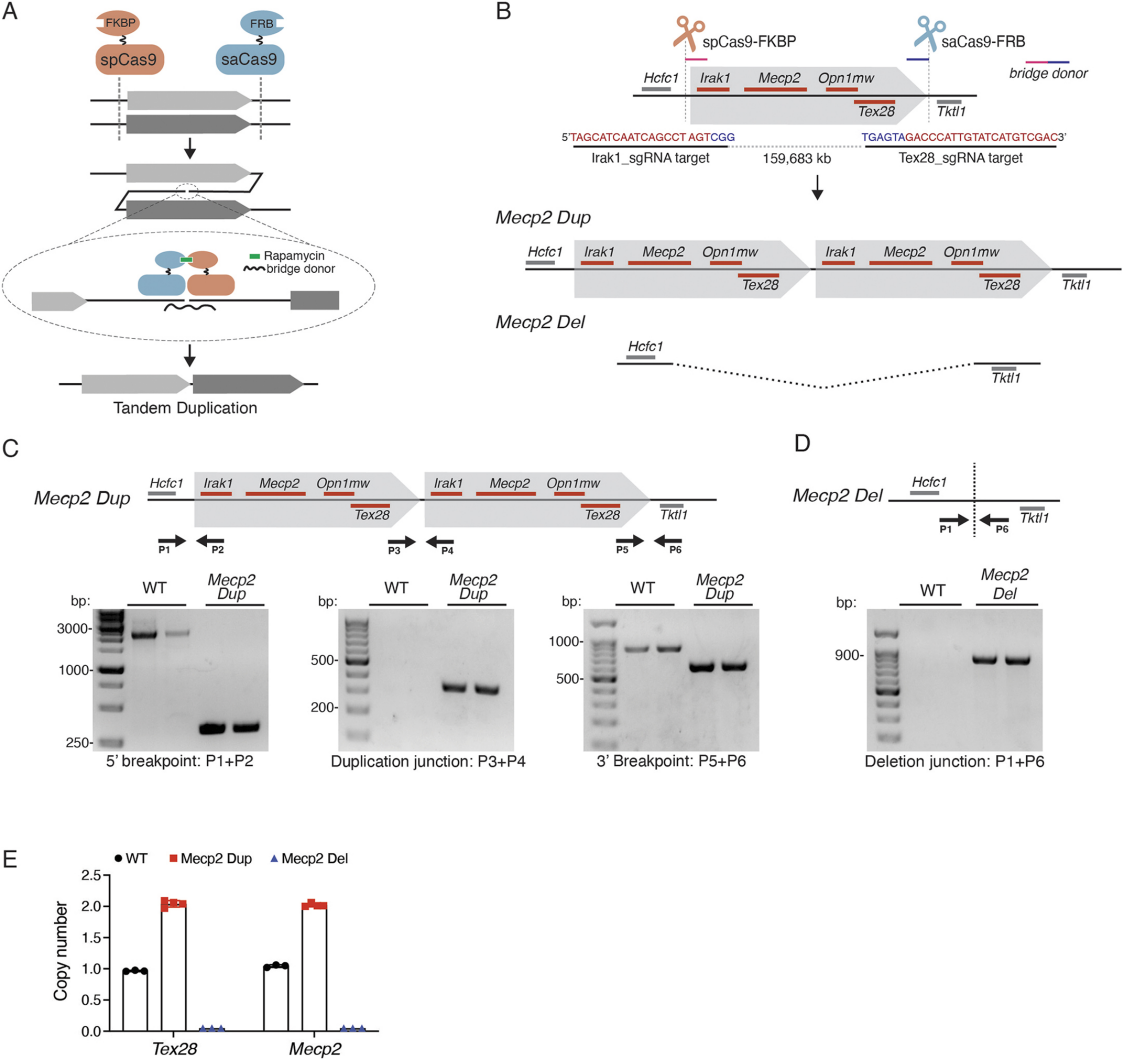
**A Cas9 fusion proximity-based approach generates the *Mecp2 Dup* and *Mecp2 Del* mouse models.** (A) Schematics of the proximity-based approach to generate tandem duplications. Two orthogonal Cas9 enzymes fused with the FK506 binding protein-12 (FKBP) (SaCas9-FKBP) and FKBP-rapamycin-binding (FRB) domain of mTOR (SpCas9-FRB) target the 3′ and 5′ ends of the region to duplicate. Upon Cas9-mediated double-stranded breaks at both target sites, the cell machinery activates the DNA damage response to ensure proper DNA repair. This process can give rise to structural variants of the region of interest, including tandem duplications. In the newly developed proximity-based approach, rapamycin treatment causes the dimerization of the FKBP and FRB domains, bringing in close proximity the two Cas9 and the free DNA ends to rearrange, increasing the probability of tandem duplication generation. A double-stranded donor (bridge donor) bridging the duplication junction is added together with the CRISPR/Cas9 components to promote the occurrence of a precise junction. (B) An ∼160 kb region encompassing the genes *Irak1*, *Mecp2*, *Opn1mw* and *Tex28* was targeted to generate the *Mecp2 Dup* and *Mecp2 Del* mouse models. The target sequences for both guides used are shown, with the PAM sequences denoted in blue and the guide sequence in red. Cas9 and the sgRNAs are represented as scissors. The bridge donor overlapping sequences are presented in pink (5′ sequence: 136 bp upstream *Irak1*_sgRNA target site) and blue (3′ sequence:186 bp downstream *Tex28*_sgRNA target site). (C) PCR amplification of the duplication 5′ and 3′ breakpoints and junction in the *Mecp2 Dup* founder mouse, confirmed by whole-genome sequencing. Primers are represented by arrows. (D) PCR amplification of the deletion junction in the *Mecp2 Dup* founder mouse. Primers are represented by arrows. (E) Digital droplet PCR confirmed the copy number of the duplicated or deleted region with probes targeting *Tex28* and *Mecp2.* A probe targeting the *Tfrc* gene was used as reference.

It is essential to optimize current strategies to generate mouse models recapitulating disease-causing structural genomic variants, including large deletions and, in particular, tandem duplications. We hypothesized that the efficiency of CNV generation would be enhanced by promoting the physical proximity of the DNA ends involved in the rearrangement. The approach we developed utilizes the ability of the FK506 binding protein-12 (FKBP) and FKBP-rapamycin-binding (FRB) domains to dimerize upon rapamycin binding (Banaszynski et al., 2005). Our system consists of two orthogonal Cas9 enzymes targeting the 3ʹ and 5′ ends of the region of interest (Fig. 1A). A *Staphylococcus aureus* (SaCas9) is fused with FKBP (SaCas9-FK506), paired with a single-guide RNA (sgRNA) targeting the 3′ region of the desired rearrangement. Concurrently, *Streptococcus pyogenes* Cas9 (SpCas9) is fused with the FRB domain of mTOR (SpCas9-FRB), with a sgRNA targeting the 5′ region. Upon rapamycin treatment, the FKBP and FRB domains dimerize, bringing the two Cas9 proteins and the free DNA ends in close proximity, and promoting the occurrence of the desired CNV. To favor the formation of a precise duplication junction, a double-stranded DNA PCR product, referred to as the bridge donor, spanning the duplication junction was added to the system. Furthermore, we reasoned that performing the above-described genetic modification during the long G2 phase of the cell cycle of two-cell-stage mouse embryos would improve the efficiency of tandem duplication, due to the presence of closely aligned sister chromatids (Gu et al., 2018).

We applied this methodology to generate a mouse model with the intent to generate a tandem duplication mimicking the minimal mutations causing MDS in patients, spanning the genes *IRAK1* and *MECP2*. We designed two sgRNAs, referred to as Irak1_sgRNA and Tex28_sgRNA, flanking the ∼160 kb target region for duplication, which includes the genes *Irak1*, *Mecp2*, *Opn1mw* and *Tex28* (chrX: 74,012,497-74,172,198) (Fig. 1B). Despite not being included in the minimal duplicated region found in patients, the genes *Opn1mw* and *Tex28* were included in the duplicated region due to the presence of repetitive sequences that hindered the design of specific sgRNAs flanking the *Mecp2* gene.

We microinjected Cas9 mRNAs, sgRNAs and the 331 bp bridging donor into 180 two-cell-stage CD-1 mouse embryos, following the previously established protocol (Gu et al., 2018). Embryos were then treated with rapamycin for 6 h to allow dimerization and then transferred to pseudo-pregnant females. Twenty-four live-born pups were produced. The pups were screened using PCR amplifying the putative tandem duplication junction. As a two-guide approach is a well-established technique to generate genomic deletions, we also screened the newborn mice for the presence of the reciprocal *Irak1-Tex28* deletion. The tandem duplication and deletion generation efficiency observed were 3/24 (12%) and 16/24 (66%), respectively. Out of three founder mice, only one mouse transmitted the correct tandem duplication to the progeny. This founder mouse showed the presence of both a tandem duplication and a large deletion, suggesting reciprocal *Irak1-Tex28* duplication/deletion. This mouse was outcrossed to wildtype (WT) mice to establish the *Mecp2 Dup* and *Mecp2 Del* mouse lines, respectively, harboring a *Irak1-Tex28* duplication and deletion.

Given that CRISPR/Cas9 may generate mosaic mutations, we conducted comprehensive genetic quality control in N1 progenies as described previously (Gu et al., 2022). Because inadvertent structural variations may be generated by the editing process, the *Mecp2 Dup* N1s were analyzed by whole-genome sequencing (WGS), which confirmed the presence of the intact tandem duplication. Minimal rearrangements were identified at both the duplication junction and breakpoints, which represent the guide target sites (Table S1). No rearrangements were detected at the top ten off-target sites for each sgRNA (Tables S2 and S3) and no non-specific integration of the bridging donor was observed in the WGS analysis. PCR followed by Sanger sequencing confirmed a 2026 bp deletion around the Irak1_sgRNA target site and a 252 bp deletion at the Tex28_sgRNA target site (Fig. 1C; Fig. S1B,C). The duplication junction harbors a 170 bp insertion compared with the predicted junction (Fig. S1A). These minor insertions/deletions are considered not to impact the expression of the primary MDS-defining genes *Mecp2* or *Irak1*, as they do not encompass known gene expression regulatory sequences, such as the miRNA146a-binding site in the 3′ untranslated region of *Irak1* (Zhou et al., 2019), and the noncoding *cis*-regulatory elements identified in the region surrounding the *Mecp2* locus (Shao et al., 2021b)*.* Furthermore, genetic changes in patients with MDS represent non-recurrent duplications with inconsistent junction sequences; thus, these minor changes mimic the genetic landscape found in patients with MDS and are unlikely to affect the phenotypes of our mouse model. PCR followed by Sanger sequencing was used to validate the presence of the predicted deletion junction in the *Mecp2 Del* mouse model (Fig. 1D; Fig. S1D). Digital droplet PCR (ddPCR) confirmed the predicted copy number of the region in *Mecp2 Dup*, *Mecp2 Del* and WT control mice (Fig. 1E).

The deletion of *Mecp2* resembles the *MECP2* loss-of-function variants seen in the context of RTT. Accordingly, *Mecp2 Del* mice developed a phenotype compatible with RTT including a reduced lifespan, compromised motor coordination and clasping behavior (Fig. S2A-D). In summary, our Cas9 fusion proximity-based approach generated the first tandem duplication mouse model of MDS, coined the *Mecp2 Dup* mouse, as well as an RTT mouse model known as the *Mecp2 Del* mouse, which was used as a control for our experiments.

### The *Mecp2 Dup* mouse model exhibits increased *Mecp2* and *Irak1* expression

**Fig. 2.**
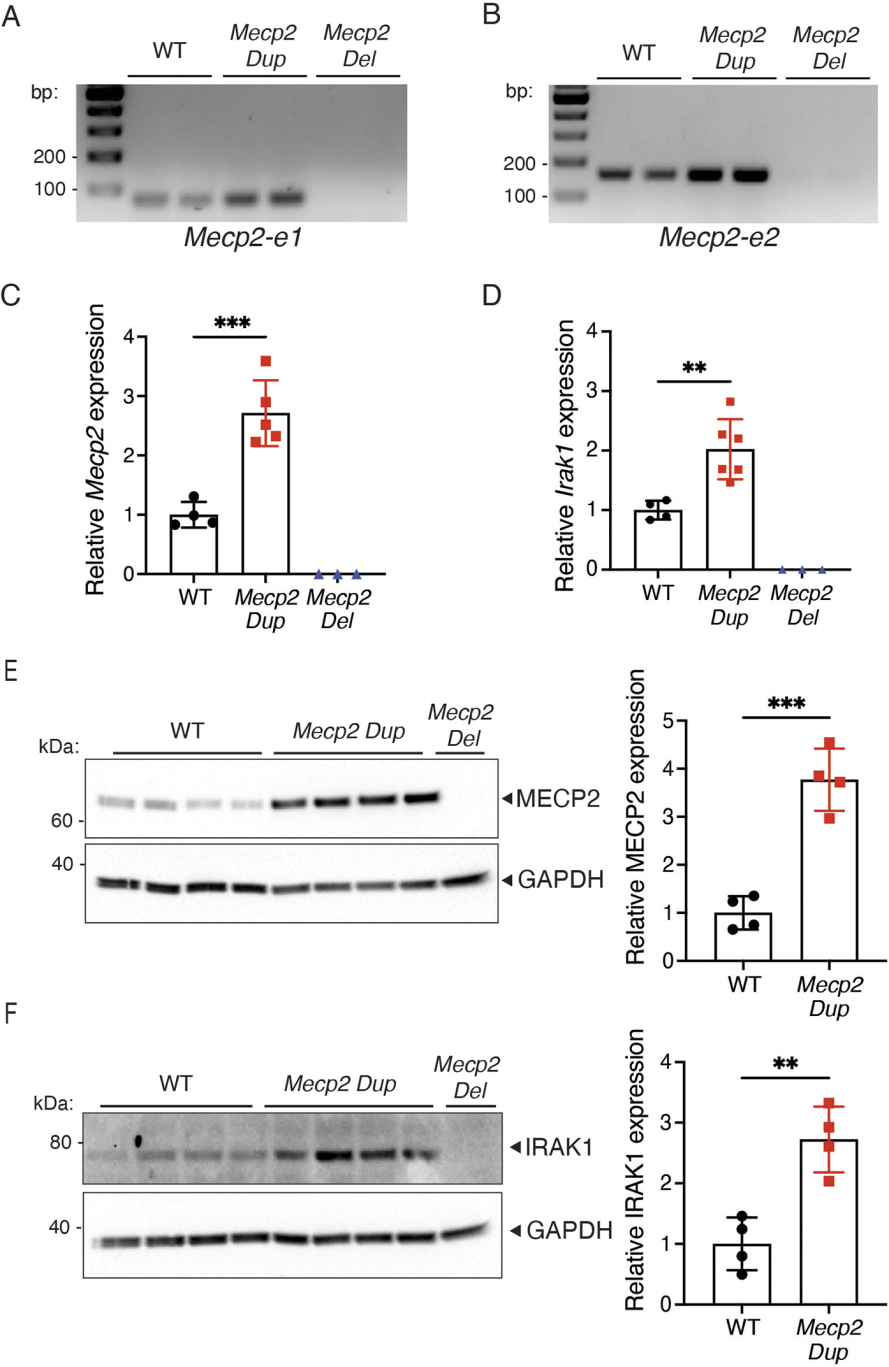
***Mecp2* and *Irak1* are overexpressed in the hippocampus of *Mecp2 Dup* mice.** (A,B) Reverse transcription PCR analysis detected (A) *Mecp2-e1* and (B) *Mecp2-e2* expression in the hippocampus of WT and *Mecp2 Dup* mice. No *Mecp2* was observed in the hippocampi of *Mecp2 Del* mice. (C) The levels of *Mecp2* expression were analyzed via qPCR in 10-week-old *Mecp2 Dup* mice, WT littermates and *Mecp2 Del* mice. The data were normalized over *Gapdh* expression. WT, *n*=4; *Mecp2 Dup*, *n*=5; *Mecp2 Del*, *n*=3. *P*=0.0007. (D) *Irak1* transcript levels were analyzed via qPCR. The data were normalized over *Gapdh* expression. WT, *n*=4; *Mecp2 Dup*, *n*=6; *Mecp2 Del*, *n*=3. *P*=0.0048. (E,F) Western blot analysis confirmed increased (E) MECP2 and (F) IRAK1 expression in the hippocampus of *Mecp2 Dup* mice compared to that in WT littermates. GAPDH served as a loading control (left). Densitometry analysis to quantify the amount of MECP2 (*P*=0.0003) and IRAK1 (*P*=0.0026) expression (right). WT, *n*=4; *Mecp2 Dup*, *n*=4; *Mecp2 Del*, *n*=1. All data are represented as the mean±s.d. Statistical analyses were performed with two-tailed unpaired Student's *t*-test. **P<0.01; ***P<0.001.

Given that the *Mecp2* gene through alternative splicing produces two distinct isoforms, denoted as *Mecp2-e1* and *Mecp2-e2*, each presenting with unique N-terminal regions, temporal expression patterns and functions (Martínez de Paz et al., 2019), we conducted an analysis of their expression in the *Mecp2 Dup* mouse model. Transcript analysis confirmed the expression of both *Mecp2-e1* and *Mecp2-e2* isoforms in the brains of *Mecp2 Dup* mice (Fig. 2A,B). To evaluate the levels of *Mecp2* expression, we performed quantitative real-time PCR (qRT-PCR) in various brain areas, including the hippocampus, cortex and cerebellum, identifying a 2- to 2.7-fold increase in expression compared to that in WT littermates across all of the regions analyzed (Fig. 2C; Fig. S3A). *Irak1* expression was increased by 2- to 2.3-fold in the brain of *Mecp2 Dup* mice compared with that in WT controls (Fig. 2D; Fig. S3B). Transcript analysis confirmed the absence of both *Mecp2* and *Irak1* expression in the brain of *Mecp2 Del* mice (Fig. 2C,D). MECP2 and IRAK1 protein levels were evaluated in the aforementioned brain areas via immunoblotting, with values correlating with the transcript levels for both MECP2 and IRAK1, ranging from 2.3- to 3.7-fold and 1.8- to 2.7-fold compared to levels in WT mice, respectively (Fig. 2E,F; Fig. S3C-H). The levels of *Tex28* and *Opn1mw* transcripts, predominantly found in the testis and retina, respectively, were assessed using quantitative PCR (qPCR) in their respective key tissues. No significant upregulation of either gene was observed in *Mecp2 Dup* mice compared to its expression in controls, even if a trend towards increased expression was observed for *Opn1mw* (Fig. S3I,J). Consequently, further investigation into the involvement of these genes in the disease phenotypes was not pursued.

### *Mecp2 Dup* mice develop a progressive neurological phenotype

**Fig. 3.**
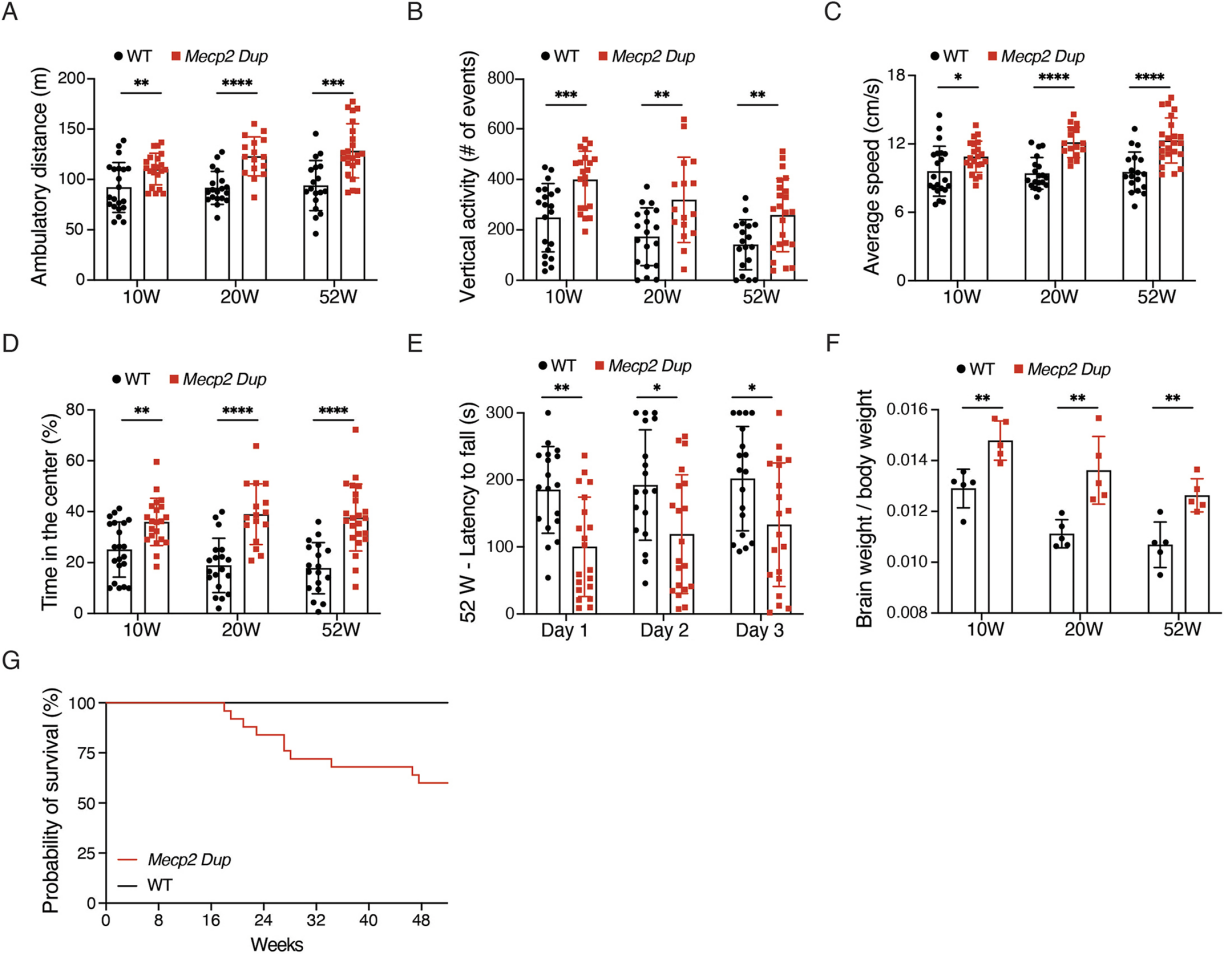
***Mecp2 Dup* mice develop progressive neurological phenotypes.** *Mecp2 Dup* mice showed increased activity and reduced anxiety-related behaviors on the open-field test compared to those observed for WT littermates at 10, 20 and 52 weeks of age. The parameters measured included (A) ambulatory distance, (B) vertical activity, (C) average speed, (D) time spent in the central area of the arena (10 weeks, *P*=0.0012; 20 weeks, *P*=8.4878×10^−6^; 52 weeks, *P*=4.6469×10^−6^). *Mecp2 Dup*, *n*=16-23; WT, *n*=18-21. Statistical analyses were performed with two-tailed unpaired Student's *t*-test. (E) A 3-day accelerated rotarod test identified reduced motor coordination in *Mecp2 Dup* mice at 52 weeks of age. *Mecp2 Dup*, *n*=20; WT, *n*=18. Data were analyzed by two-way ANOVA repeated measures followed by Bonferroni's multiple comparison test. (F) Brain-to-body weight ratio was measured at every time point. *Mecp2 Dup*, *n*=5; WT, *n*=5. Statistical analyses were performed with two-tailed unpaired Student's *t*-test. (G) Survival analysis of *Mecp2 Dup* mice up to 52 weeks of age was reduced compared to WT littermates (*P*=0.0084). *Mecp2 Dup*, *n*=23; WT, *n*=13. Survival curves were compared with the Mantel–Cox test. All data are represented as the mean±s.d. *P<0.05; **P<0.01; ***P<0.001; ****P<0.0001.

We evaluated the activity of *Mecp2 Dup* mice and WT littermates at 10, 20 and 52 weeks of age using the open-field test. Starting at 10 weeks, *Mecp2 Dup* mice showed increased activity in the open-field test for all the parameters analyzed, including total distance traveled, vertical activity and average speed, with symptoms progressing as the mice aged. At 10 weeks of age *Mecp2 Dup* mice showed a 20% increase in total distance traveled versus that for WT mice (*P*=0.0068), 60% increase in vertical activity (*P*=0.0003) and 13% increase in average speed (*P*=0.0297) in the open-field arena (Fig. 3A-C). At 20 weeks of age, these parameters increased up to 35% (*P*=1.1461×10^−5^), 84% (*P*=0.0047) and 29% (*P*=1.4812×10^−6^), respectively, and remained stable up to 52 weeks of age (Fig. 3A-C). In addition, the *Mecp2 Dup* mice spent up to twice the amount of time in the center of the open-field arena compared with that spent by WT littermates at every time point analyzed (Fig. 3D). These data suggest that *Mecp2 Dup* mice display reduced anxiety compared to WT mice. In support of this observation, we also observed abnormal levels of activity in the center of the open-field arena, where MDS mice traveled 1.5-2.2 times more distance compared to that traveled by WT mice, and showed 2.3-4.5 times more vertical activity (Fig. S4A,B). *Mecp2 Dup* mice also spent 40-50% less time resting in the periphery of the open-field arena (Fig. S4C). We used a 3-day accelerated rotarod test to analyze motor coordination and cerebellar learning at 10, 20 and 52 weeks of age. Minimal differences were observed at 10 and 20 weeks of age (Fig. S5A,B). However, at 52 weeks, the *Mecp2 Dup* mice showed reduced motor coordination compared with that of WT littermates, falling from the rotarod 37-48% (day 1, *P*=0.0048; day 2, *P*=0.0184; day 3, *P*=0.0301) earlier each day of the test (Fig. 3E). No significant differences in body weight of MDS mice and WT controls were detected up to 38 weeks of age, when the *Mecp2 Dup* mice became significantly lighter (Fig. S6A). Notably, *Mecp2 Dup* mice showed a 15-22% increase in brain-to-body weight ratios (10 weeks, *P*=0.0046; 20 weeks, *P*=0.0047; 52 weeks, *P*=0.0043) at each time point analyzed (Fig. 3F). The *Mecp2 Dup* mice had a reduced life span (Mantel–Cox test, *P*=0.0084), with 40% mortality between 18 and 52 weeks of age, compared to WT littermates, for which no deaths were observed (Fig. 3G).

### *Mecp2 Dup* mice develop hippocampal-related phenotypes

**Fig. 4.**
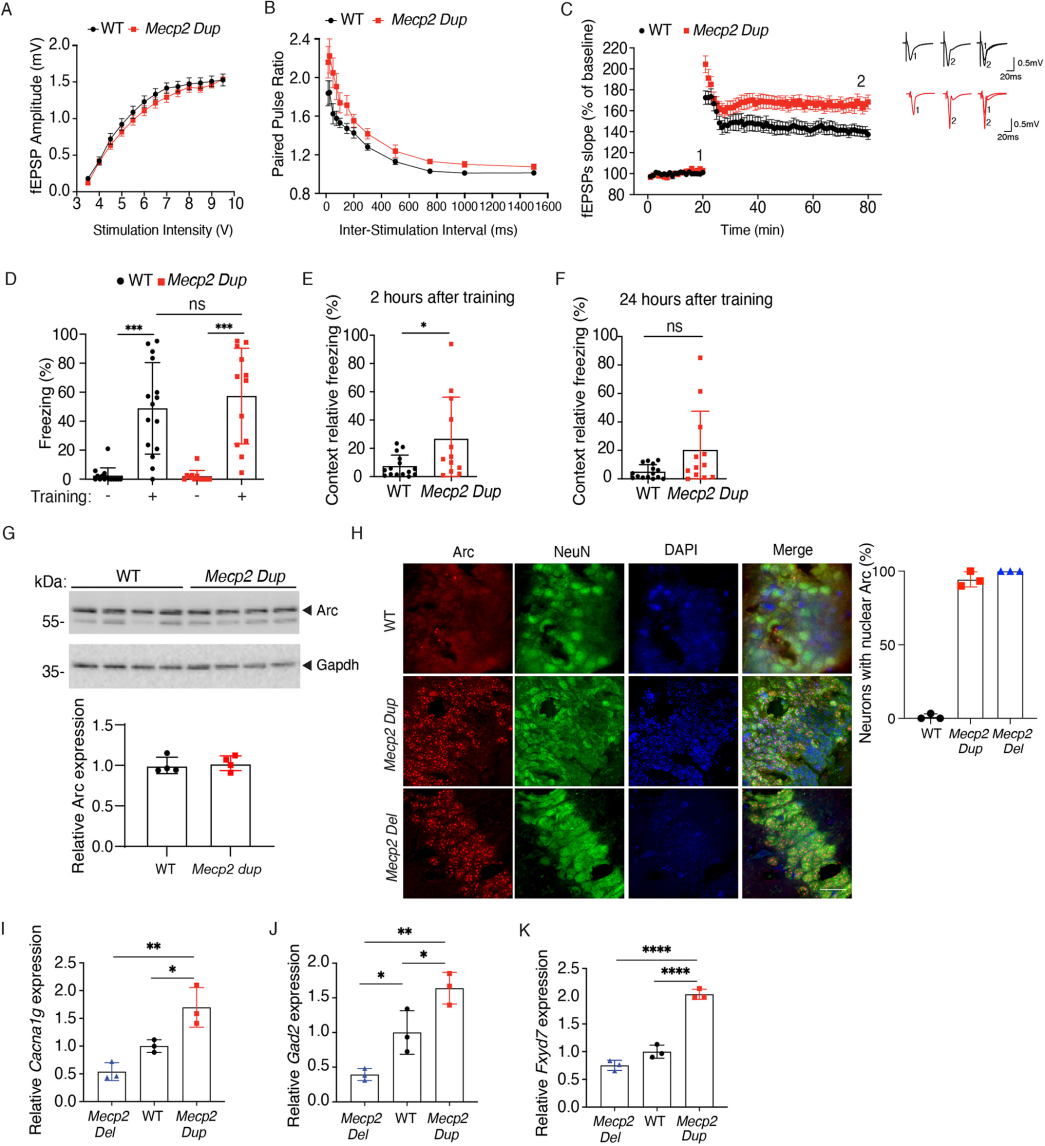
**Electrophysiology analysis and fear conditioning test show abnormalities in hippocampal function in *Mecp2 Dup* mice.** (A) Scatterplot of field excitatory postsynaptic potential (fEPSP) peak amplitudes as a function of stimulus intensity for each genotype. *Mecp2 Dup*, *n*=17; WT, *n*=17. (B) Scatterplot of the paired pulse ratio as a function of the inter-burst interval for each genotype. *Mecp2 Dup* mice showed increased paired pulse ratio at interstimulus intervals of 15 ms (*P*<0.05), 25 ms (*P*<0.001), 50 ms (*P*<0.001) and 75 ms (*P*<0.001). *Mecp2 Dup*, *n*=11; WT, *n*=8. (C) Scatterplot of the normalized fEPSP slope over time induced by theta burst stimulation of the Schaffer collateral inputs in hippocampal slices from each genotype. Traces from each genotype are represented as an average of fifteen consecutive fEPSPs recorded at the indicated times (time points 1 and 2). The magnitude of synaptic potentiation was as follows: WT (140.9±4.9%, *n*=17) and *Mecp2 Dup* (166.3±6.5%, *n*=15). *P=*0.004. (D) In conditioned fear analysis, there was no statistical difference in response to training at 20 weeks of age between *Mecp2 Dup* mice and WT littermates. *Mecp2 Dup*, *n*=12; WT, *n*=15. (E) The percentage of freezing was enhanced in *Mecp2 Dup* mice compared to that in WT mice when tested on contextual fear conditioning 2 h after training. *Mecp2 Dup*, *n*=12; WT, *n*=15. (F) Contextual fear conditioning test performed 24 h after training showed no statistical differences for *Mecp2 Dup* mice compared to WT mice (*P*=0.1223). This suggests that *Mecp2 Dup* mice have enhanced short-term memory, whereas long-term memory is similar to that of WT mice. *Mecp2 Dup*, *n*=12; WT, *n*=15. (G) Western blot analysis indicated no difference in Arc levels in *Mecp2 Dup* mouse hippocampi compared to those of WT littermate hippocampi. GAPDH served as a loading control. Densitometry analysis is reported in the plot below the gel. (H) Immunofluorescence staining of Arc, NeuN and DAPI in WT, *Mecp2 Dup* and *Mecp2 Del* mouse hippocampi (right). Scale bar: 30 µm. Quantification of the percentage of Arc^+^ nuclear staining is reported on the left. (I-K) qRT-PCR to evaluate the expression levels of (I) *Cacna1g*, (J) *Gad2* and (K) *Fxyd7* in *Mecp2 Dup* (*n*=3), WT (*n*=3), and *Mecp2 Del* (*n*=3) mice. Statistical analysis was performed with two-way ANOVA followed by Sidak multiple comparisons test (A,B), two-tailed unpaired Student's *t*-test (C,E), Kruskal–Wallis test followed by Dunn's multiple comparison test (D), Mann–Whitney *U*-test (F) or one-way ANOVA followed by Tukey's post hoc test (I-K). Data are represented as mean±s.e.m. (A-C,G,H) or mean±s.d. (D-F,I-K). *P<0.05; **P<0.01; ***P<0.001; ****P<0.0001.

To evaluate hippocampal-related phenotypes in the *Mecp2 Dup* mice, we investigated neurotransmission at the Schaffer collateral synapses in the CA1 region of the hippocampus. To determine whether the overexpression of *Mecp2* might affect basal synaptic transmission, we recorded field excitatory postsynaptic potentials (fEPSPs) and found that the stimulation intensity–response relationship did not differ between *Mecp2 Dup* mice and WT littermates (Fig. 4A). The *Mecp2 Dup* mice showed enhanced short-term synaptic plasticity compared to that of WT littermates as determined by paired-pulse facilitation of the fEPSPs (Fig. 4B).

Moreover, we evaluated the capacity of Schaffer collateral synapses to elicit long-term potentiation (LTP). *Mecp2 Dup* mice showed a robust LTP enhancement immediately after theta burst stimulation (TBS), which persisted for at least 60 min (Fig. 4C), indicating increased synaptic plasticity compared to that of WT littermates. To determine the possible impact of these findings on hippocampal learning, we performed fear conditioning analysis in 20-week-old mice. No differences were observed in the freezing response during training between the two genotypes (Fig. 4D). We then tested the mice 2 and 24 h after training both with contextual and cued tests. *Mecp2 Dup* mice showed increased contextual freezing, presenting 3.6 (*P*=0.0219) and 4 times (*P*=0.1223) more freezing than WT mice at 2 and 24 h, respectively (Fig. 4E,F), suggesting that *Mecp2 Dup* mice have enhanced short-term memory, whereas long-term memory is similar to that of WT mice. Increased freezing behavior, albeit not statistically significant (2 h, *P*=0.5619; 24 h, *P*=0.0650), was noted on the cued test as well (Fig. S7A,B).

We also examined the abundance of Arc (also known as Arg3.1) in WT, *Mecp2 Dup* mice and *Mecp2 Del* mice. Arc is a key player in maintaining homeostatic synaptic plasticity, critical for LTP and depression of synaptic transmission. The hippocampus of the *Mecp2 Dup* mice displayed Arc levels comparable to those of WT mice (Fig. 4G). Notably, immunofluorescence staining indicated substantial increase in nuclear localization of Arc in the hippocampal neurons of *Mecp2 Dup* and *Mecp2 Del* mice; 94.4% and 100% of neurons displayed Arc nuclear staining, respectively. On the contrary, only ∼1% of WT hippocampal neurons showed Arc nuclear staining. These findings suggest a crucial role of *Mecp2* gene dosage in regulating Arc localization (Fig. 4H).

As MECP2 is also known as a global transcriptional regulator for hippocampal genes (Chahrour et al., 2008), we analyzed the expression levels of selected genes known to be dysregulated in other *MECP2* duplication models as well with qRT-PCR for *Mecp2 Dup*, *Mecp2 Del* and WT littermates at 6 weeks of age (Fig. 4I-K). We identified an increased hippocampal expression of glutamate decarboxylase 2 (*Gad2*) (*P*=0.0330), encoding an enzyme involved in γ-amino-butyric acid (GABA) synthesis; increased expression of voltage-gated calcium channel subunit α-1G (*Cacna1g*) (*P*=0.0254); and increased expression of FXYD domain-containing ion transport regulator 7 (*Fxyd7*) (*P*<0.0001), a modulator of Na^+^/K^+^-ATPase activity (Meyer et al., 2020).

### *Mecp2 Dup* mice demonstrate an abnormal immune response with elevation in pro-inflammatory and Th1-associated cytokines and chemokines

Recurrent severe respiratory tract infections are a leading cause of morbidity and mortality among patients with MDS, although the mechanisms underlying this phenomenon are not completely understood. We infected mice with the influenza H1N1 strain and assessed for immune abnormalities that would explain the heightened sensitivity of patients with MDS to such infections.

**Fig. 5.**
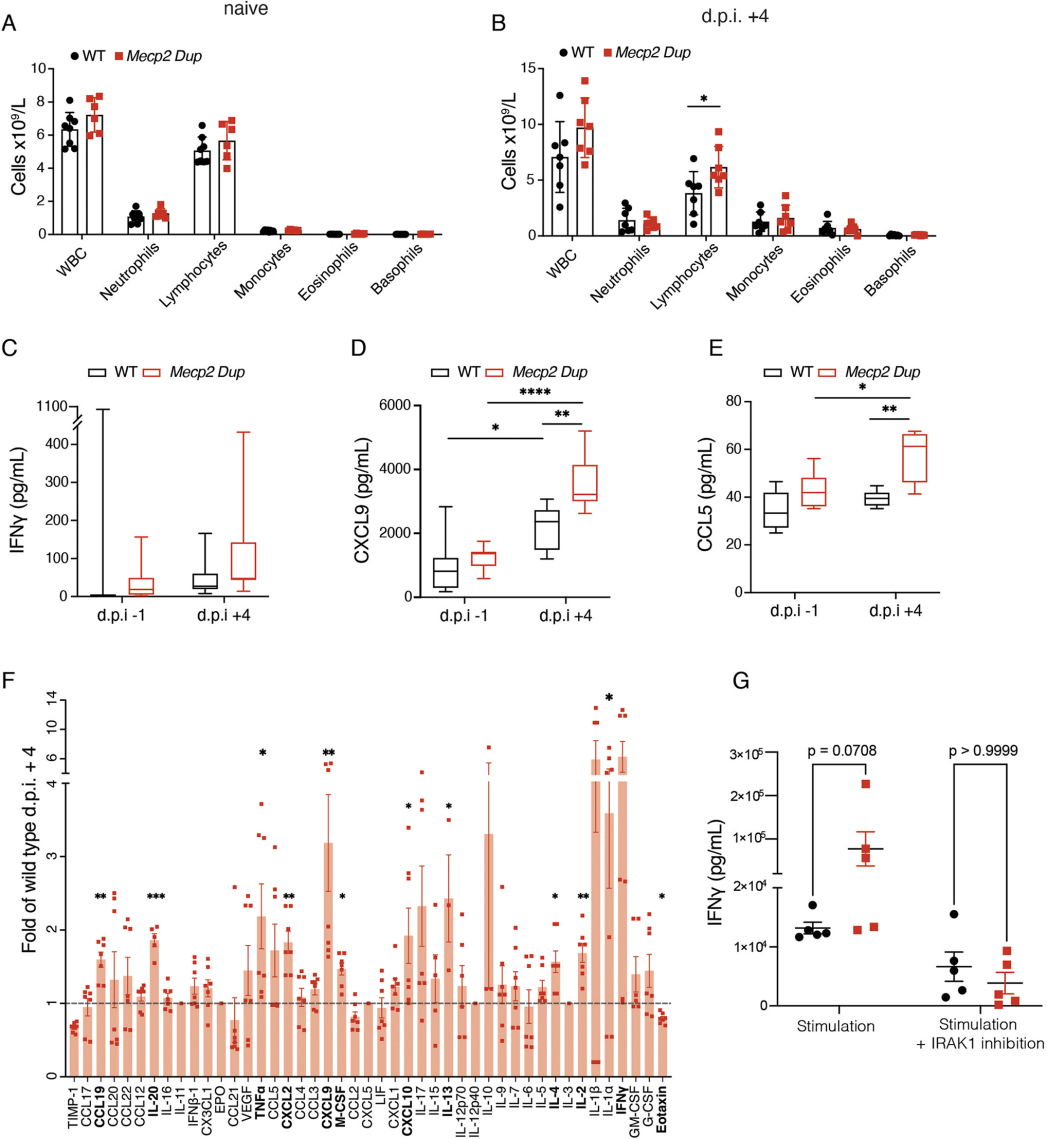
**Abnormal immune response after influenza virus infection in *Mecp2 Dup* mice.** (A) White blood cell (WBC) count in naïve mice. *Mecp2 Dup*, *n*=6; WT, *n*=8. (B) White blood cell count on day 4 after influenza infection (dpi). *Mecp2 Dup*, *n*=7; WT, *n*=7. Data are represented as the mean±s.d. Statistical analyses were performed with two-tailed unpaired Student's *t*-test. (C-E) Chemokine and cytokine multiplex analysis in the serum one day before infection (−1 dpi) and on day 4 after influenza virus infection (+4 dpi) showed elevated production of (C) IFNγ, (D) CXCL9 and (E) CCL5. Data are represented as box plots: boxes show the interquartile range, whiskers show the 5-95th percentile and the median is marked with a line. Data were analyzed by one-way ANOVA followed by Tukey’s post hoc test or Kruskal–Wallis test followed by Dunn's multiple comparisons test. (F) Cytokines and chemokines were measured in BALF. Data are presented as fold change versus WT levels and bars show the mean±s.e.m. *Mecp2 Dup*, *n*=7; WT, *n*=7. Statistical analyses were performed with two-tailed unpaired Student's *t*-test. (G) The levels of IFNγ were measured in the supernatant of splenic CD4T cells stimulated with anti-CD3, anti-CD28 and IL-18, and cultured in the presence or absence of a selective IRAK1 inhibitor. Data were analyzed by two-way ANOVA followed by Bonferroni multiple comparisons test and are represented as the mean±s.e.m. *P<0.05; **P<0.01; ***P<0.001; ****P<0.0001.

Mortality rates up to 14 days from infection were no different between *Mecp2 Dup* (28.6%) and WT (14.3%) mice (*P*=0.5302) (Fig. S8A). Body weight loss was also no different between *Mecp2 Dup* and WT mice (Fig. S8B). Complete blood count and differential, and measurement of serum cytokines and chemokines were performed 1 day prior to infection [or −1 day post infection (dpi)] and at +4 dpi (Fig. 5A-E). Evaluation of cytokines and chemokines from bronchoalveolar lavage fluid (BALF) was performed at +4 dpi (Fig. 5F).

At baseline, no notable differences were found between the blood cell counts or serum cytokines/chemokines of *Mecp2 Dup* and WT mice (Fig. 5A-E; Table S4). However, at +4 dpi, peripheral blood lymphocytes were increased almost 2-fold in *Mecp2 Dup* mice (*P*=0.0401) (Fig. 5B). Additionally, at +4 dpi, elevated serum CCL5 (1.4-fold, *P*=0.0004) and CXCL9 (1.7-fold, *P*<0.0001) were noted in *Mecp2 Dup* mice (Fig. 5D,E; Table S5). Although IFNγ levels were elevated by 2.23-fold in *Mecp2 Dup* mice, this was not statistically significant (*P*>0.999) (Fig. 5C; Table S5). More robust alterations were identified in BALF, including increased levels of TNFα (2.19-fold, *P*=0.0291), IFNγ (6.26-fold, *P*=0.0319), CXCL9 (3.19-fold, *P*=0.0088), CXCL10 (1.92-fold, *P*=0.0486), the lymphocyte-recruiting chemokine CCL19 (1.60-fold, *P*=0.0016) and IL-2 (1.69-fold, *P*=0.0062), which promotes T-cell proliferation (Fig. 5F; Table S6).

### IRAK1 inhibition diminishes differences in IFNγ secretion between *Mecp2 Dup* and WT splenocytes following *in vitro* stimulation

We previously identified an increase in IFNγ in *Mecp2 Dup* BALF following influenza infection. To determine whether abnormal IFNγ secretion in MDS mice was mediated by IRAK1, we isolated splenic CD4T cells from *Mecp2 Dup* and WT littermates and cultured them *in vitro* with anti-CD3 and anti-CD28 antibodies, as well as IL-18 (100 ng/ml), to induce robust IFNγ secretion. Cells were cultured in the presence or absence of a selective IRAK1 inhibitor. After 72 h, there was a trend toward increased levels of IFNγ in the supernatant of stimulated *Mecp2 Dup* cells cultured without an inhibitor where *Mecp2 Dup* cells showed a 4.7-fold (*P*=0.0743) increase in IFNγ compared to IFNγ levels in WT cells (Fig. 5G; Table S7). The addition of an IRAK1 inhibitor abrogated any differences in IFNγ levels between genotypes (*P*=0.9678) (Fig. 5G; Table S7).

## DISCUSSION

Modeling of complex genomic rearrangements remains a limiting factor in the study of structural genomic variants that cause diseases in humans, of which MDS is a prime example. The current study showed the implementation of the Cas9 fusion proximity-based approach to successfully and efficiently generate an MDS mouse model harboring a disease-defining 160 kb tandem duplication involving both *Mecp2* and *Irak1* (*Mecp2 Dup*) and its reciprocal deletion mouse model (*Mecp2 Del*). *Mecp2 Dup* mice demonstrated abnormal neurodevelopmental features in keeping with patient phenotypes as well as existing MECP2 overexpression models. Moreover, *Mecp2 Dup* displayed evidence for immune aberrations in keeping with enhanced inflammation and IFNγ activation, possibly owing to the contribution of heightened IRAK1 expression not previously investigated in the context of the disease.

Efficient modeling of disease-causing CNVs is vital for the investigation of and therapeutic development in the context of rare inherited diseases. The current CRISPR/Cas9 dimerization approach increased the frequency of generating tandem duplications to 12% in mouse embryos, compared to the 1-3% efficiency previously observed by our group and others (Kraft et al., 2015; Maino et al., 2021; Pristyazhnyuk et al., 2019). This is likely due to the physical proximity of the rearranged DNA ends upon rapamycin treatment. This hypothesis is supported by previous work showing that binding of modified Cas9 to repair templates to ensure vicinity to the insertion site increased knock-in efficiencies (Gu et al., 2018; Ma et al., 2017; Savic et al., 2018). However, a more in-depth analysis would be needed to dissect the direct effect of the physical proximity on the duplication generation efficiency. Previous published studies investigated efficiencies of CNV generation by editing mouse embryonic stem cells (Kraft et al., 2015; Maino et al., 2021), zygotes (Boroviak et al., 2016; Hara et al., 2016; Korablev et al., 2017) or one-cell-stage embryos (Li et al., 2015). Instead, we performed the microinjection of the Cas9 mRNA and sgRNAs in two-cell-stage embryos that are known for having a longer G2 phase and open chromatin structure, and have previously been used to increase knock-in efficiencies (Gu et al., 2018). Moreover, we speculate that having two sister chromatids available might be critical in the case of duplication generation, where the second chromatid acts as a template for the generation of the tandem duplication. However, the effect of this method and the necessity of bringing the two ends of duplication to proximity is expected to be locus dependent. In particular, the effects can be significantly impacted by three-dimensional chromatin structure and locus–locus interaction frequencies in a particular embryo stage, such as zygotes or two-cell-stage embryos. More systematic testing for generating multiple duplication models is needed to establish whether this method is superior to other CRISPR duplication generation methods reported before. Thus, our method provided a useful option to be applied when the traditional CRISPR technologies such as zygote or two-cell-stage embryo injection of Cas9 and dual sgRNAs face challenges.

The newly generated *Mecp2 Dup* mouse model presents several possible advantages compared with transgenic models hitherto described. These include the faithful recapitulation of the genomic rearrangement seen in patients with MDS, expression of *Mecp2* under its endogenous promoter, consequent expression of the two different transcript isoforms of the gene, and the concurrent duplication and overexpression of *Irak1*. The *Mecp2 Dup* mouse model exhibits neurodevelopmental phenotypes consistent with observations in patients and other transgenic mice. Notably, as seen in other MDS models, hippocampus-related phenotypes are prominent in *Mecp2 Dup* mice. Our model displays enhanced LTP and increased contextual freezing behavior, indicative of abnormal synaptic plasticity and learning, following the same behavioral presentations observed in *MECP2-Tg* and *hDup* mouse models (Collins et al., 2004; Shao et al., 2021a; Sztainberg et al., 2015).

One possible reason for the enhanced LTP is a weakened synaptic downscaling, which is a negative feedback mechanism to reduce synaptic strength in potentiated neurons (Siddoway et al., 2014). Arc is one of the critical players in downscaling synaptic strength (Suzuki et al., 2020). Notably, germline *Arc* knockout (KO) mice also exhibit an enhanced LTP magnitude and prolonged maintenance after TBS (Kyrke-Smith et al., 2021). This leads us to postulate that in *Mecp2 Dup* mice, the retention of Arc in the nucleus may impair its cytoplasmic function related to AMPA receptor endocytosis (Chowdhury et al., 2006; Shepherd et al., 2006). On another note, chronic LTP induction by 8-h BDNF stimulation in neurons induced Arc nuclear localization in hippocampal neurons (Korb et al., 2013), which aligns with our results associating Arc nuclear localization and chronic excitation in the neurons. The *Mecp2 Del* mice also showed nuclear localization of Arc. This is coherent with a previous report showing the accumulation of the AMPA receptor subunit GluA1 on synaptic surfaces in the *Mecp2-KO* mouse model for RTT (Li et al., 2016). The strong synaptic strength at baseline in turn prevented LTP. Yet, the mechanism of how MECP2 dosage determines the subcellular distribution of Arc remains unknown.

Furthermore, consistent with the role of MECP2 as a transcriptional regulator, we observed dysregulation of well-known MECP2 targets in the hippocampus of *Mecp2 Dup* mice compared to controls*.* Among these genes are *Gad2*, previously shown to be regulated by MECP2 in *MECP2-Tg* mice and in MECP2-deficient neurons (Chahrour et al., 2008; Chao et al., 2010); *Cacna1g*, which has recently been associated with intellectual disability and epilepsy and found to be consistently upregulated in other MDS mouse models (Berecki et al., 2020; Chemin et al., 2018; Striessnig, 2021; Chahrour et al., 2008; Samaco et al., 2012; Sztainberg et al., 2015); and *Fxyd7*, a modulator of Na^+^/K^+^-ATPase activity, consistently reported to be upregulated in the context of MDS (Chahrour et al., 2008; Samaco et al., 2012) and downregulated in RTT in *Mecp2-null* mice (Ben-Shachar et al., 2009). Notably, these targets were consistently upregulated in *Mecp2 Dup mice* and downregulated in *Mecp2 Del* mice, indicating the central role of *Mecp2* in regulating the transcription of these genes.

In late disease stages, *Mecp2 Dup* showed reduced performance and motor coordination on the rotarod test, consistent with the impairment of motor skills reported in patients with MDS (Ta et al., 2022) as well as in the *Tau-Mecp2* mouse model (Na et al., 2012). Severe motor coordination deficits are also reported in *Mecp2-Tg3* mice (Collins et al., 2004; Montgomery et al., 2018), overexpressing up to 5-fold *Mecp2* compared to WT mice, which are representative of the severe phenotypes observed in *Mecp2* triplication syndrome. Instead, other MDS mice, such as *MECP2-Tg* and *hDup* mice (Shao et al., 2021a; Sztainberg et al., 2015) show the opposite phenotype when tested on the rotarod.

Unlike patients with MDS and other MDS mouse models, our *Mecp2 Dup* mouse model exhibits hyperactivity and reduced anxiety starting in early disease stages. This phenotype is more commonly associated with intellectual disability in other mouse models, such as fragile X syndrome (Kazdoba et al., 2014). Conversely, other MDS mouse models, such as *Mecp2-TG*, *hDup* and *Mecp2-Tg3* mice, commonly display increased anxiety and reduced exploration behavior (Montgomery et al., 2018; Shao et al., 2021a; Sztainberg et al., 2015).

*Mecp2 Dup* mice exhibit an increased brain-to-body ratio, similar to patients with ASD and ASD mouse models (Courchesne et al., 2019; Deliu et al., 2018), a phenotype also reported in *Mecp2-Tg3* mice and patients having *MECP2* triplication syndrome (Montgomery et al., 2018).

Finally, consistent with observations in patients with MDS, *Mecp2 Dup* mice exhibit reduced life expectancy, with only 60% of mice reaching 52 weeks of age. A similar phenotype is observed in *Mecp2-Tg3* mice, although the severity is greater, with mice reported to die between 3 and 20 weeks of age (Collins et al., 2004; Montgomery et al., 2018). However, the reasons for the death of *Mecp2 Dup* mice have not been thoroughly investigated.

The discrepancies in MDS disease presentations between our model and existing MDS mouse lines may be attributed to several factors. Our *Mecp2 Dup* mouse model is the first to account for the minimal duplicated region shared across patients with MDS, encompassing both the *Mecp2* and *Irak1* genes. Given the complex interplay between *Mecp2* and *Irak1* (Kishi et al., 2016), as well as their observed overexpression at the mRNA and protein level in the *Mecp2 Dup* mouse, it is possible that the combined effects of these duplicated genes produce a different phenotype than that of MECP2 overexpression alone. In addition, strain-specific differences are likely to influence disease manifestations. *Mecp2 Dup* mice were generated on a CD-1 outbred background, whereas the other models were generated on inbred backgrounds such as C57BL/6 (Na et al., 2012), FVB (Collins et al., 2004), C57BL/6JOla×CBA hybrid (Koerner et al., 2018) and FVB/N×C57Bl/6 hybrid (Shao et al., 2021a; Sztainberg et al., 2015). Supporting this hypothesis are also the observations reporting *Mecp2* conferring different phenotypes when modeled on different mouse backgrounds in the context of RTT (Vashi and Justice, 2019). Overall, CD-1 outbred mice are known to better reflect the genetic diversity of the human population, but are usually less used compared to inbred strains because of increased variability in results and the need to use higher numbers of animals. Inbred and outbred mouse strains, even if presenting unique advantages and disadvantages, are both suitable to investigate mouse behavior and neurological disorders using memory and learning, sociability, cognition and stress/anxiety tests (Hsieh et al., 2017; Sultana et al., 2019). However, it has been shown that animals generated on different backgrounds exhibit different baseline behavioral patterns, suggesting that strain-specific genetic variations can influence disease manifestations, limiting the ability of comparing phenotypes modeled on different backgrounds (Sultana et al., 2019). To further dissect the neurobehavioral effects associated with the *Mecp2-Irak1* duplication and to compare these effects with those observed in existing MDS models, it would be beneficial to assess this duplication on an inbred background. This approach would provide a more direct comparison with the genetic backgrounds used in current MDS mouse lines and determine whether the observed differences are mutation specific or strain specific.

Infections are a leading cause of morbidity and mortality in patients with MDS. Recurrent infections have been reported in up to 75% of cases, with a predominance of respiratory disease caused by both viral and bacterial pathogens (Bauer et al., 2015). In this work, *Mecp2 Dup* mice showed no clear baseline differences in regard to major white blood cell subsets or serum cytokine and chemokine levels. Following influenza infection, *Mecp2 Dup* mice developed elevated peripheral blood lymphocyte counts. The role of MECP2 in lymphocyte proliferation was previously demonstrated in cultured clonal T cells from patients with RTT, where MECP2 deficiency led to a growth disadvantage and a blunted response to mitogen stimulus (Balmer et al., 2002). Work in patients with MDS previously identified an increase in naïve (CD45RA^+^) CD4^+^ T cells, possibly also indicating abnormal T cell proliferation. Importantly, memory (CD45RO^+^) CD4^+^ T cells were conversely reduced, suggestive of abnormal generation or reduced survival of memory T cells (Bauer et al., 2015; Yang et al., 2012). *Mecp2 Dup* mice also showed aberrations in serum and BALF cytokines and chemokines following influenza infection, suggestive of heightened T cell proliferation (IL-2) and tissue recruitment (CCL19), as well as Th1 skewing (IFNγ, CXCL9, CXCL10 and CCL5) and inflammation (TNFα). A subsequent *in vitro* experiment suggests that *Irak1* plays a role in the trend towards heightened IFNγ production by *Mecp2 Dup* in response to IL-18 stimulation, as IRAK1 inhibition diminished any differences in IFNγ secretion between genotypes.

Our finding of Th1 skewing of the immune response following infection shows differences compared to findings in *MECP2*-overexpressing transgenic mice (Cronk et al., 2017; Yang et al., 2012). Yang et al. (2012) reported that *Mecp2-Tg3* mice could not control an infection with the intra-macrophagic pathogen *Leishmania* and showed impaired IFNγ secretion from involved lymph nodes. Their work also demonstrated decreased ability of CD4^+^ T cells to differentiate into IFNγ-secreting cells *in vitro*. Subsequent work by Cronk et al. (2017) showed increased mortality of *Mecp2-Tg3* mice following influenza A infection, with decreased lung lymphocytes and reduced IFNγ in BALF. However, from a clinical standpoint, patients with MDS present quite differently from patients with complete or partial IFNγ pathway deficiency, as the latter typically experience predisposition to infections with mycobacteria, *Salmonella* sp. and intra-macrophagic pathogens (Bustamante et al., 2014), not seen in patients with MDS (Bauer et al., 2015). One factor likely contributing to the discrepancy between our findings and those identified in *Mecp2-Tg3* mice may be the role of IRAK1, overexpressed at both transcript and protein level in *Mecp2 Dup* mice, which is unaccounted for by previous models. IRAK1 signaling was previously shown to cause Th1 differentiation via induction of T-bet (Cai et al., 2021; Deng et al., 2003; Heiseke et al., 2015). Additionally, increased IRAK1 expression in the context of Behçet's disease was associated with increased production of IFNγ (Sun et al., 2018). Interestingly, even MECP2 itself was previously demonstrated to be essential to differentiation of naïve CD4^+^ into Th1 cells (Jiang et al., 2014), whereas loss of MECP2 expression in patients with RTT was associated with a shift away from Th1 and loss of IFNγ expression. This suggests that perhaps the precise effect of MECP2 aberrations could be dose dependent, whereby an overexpression by up to 5-fold leads to a different effect than that for a 2-fold increase. Clinically, chronic immune skewing toward Th1 has the potential to result in viral lung infections, chronic lung inflammation and impairment in immune memory, as noted, for example, in some patients with STAT1 gain of function (Scott et al., 2022). However, further testing and sampling of both serum and tissue samples from acutely infected patients with MDS, *Mecp2 Dup* mice and mice carrying the *Mecp2* and *Irak1* duplications in isolation will be required to further dissect the contribution of each gene to the disease immunophenotypes.

From a treatment standpoint, the *Mecp2 Dup* mouse model could be instrumental to the testing and development of therapeutic strategies targeting MDS. Current MDS therapies in preclinical development are mainly based on the reduction of MECP2 expression levels by using antisense oligonucleotides (ASOs) (Shao et al., 2021a). The *Mecp2 Dup* model could be a valuable model for precise titration of ASOs to avoid phenotypic conversion to RTT. In addition, more ASOs could be designed and tested to target and ameliorate overexpression of other duplicated genes such as *Irak1*. Moreover, specific gene-editing strategies such as CRISPR/Cas9-based duplication correction (Maino et al., 2021) could be tested in the *Mecp2 Dup* model to precisely correct the disease-causing variant and avoid the risk of over-targeting the duplicated genes.

In summary, our work showcases a platform to efficiently generate mouse models that faithfully recapitulate disease-causing structural variants. The newly generated *Mecp2 Dup* mouse model provides an opportunity to model the neurodevelopmental traits of patients with MDS and further explore patient predisposition to severe respiratory disease via dysregulated interferon immunity. The *Mecp2 Dup* mouse is a valuable tool to advance investigations of MDS disease mechanisms, as well as test and advance new genomic therapies for patients with MDS.

## MATERIALS AND METHODS

### Construct generation

For constructing the PCS2+ spCas9-FKBP plasmid, the FKBP coding sequence was amplified from the PM-FRB-Cerulean-T2A-FKBP-5-ptase plasmid (Addgene #40897, deposited by Dr Peter Varnai) (Tóth et al., 2012) and used to replace the monomeric streptavidin (mSA) coding sequence of the PCS2+ Cas9-mSA plasmid (Addgene #103882) (Gu et al., 2018) using infusion cloning (Takara). For construction of the PCS2+ saCas9-FRB plasmid, the saCas9 and FRB coding sequences were amplified from the pX601 plasmid (Addgene #61591, deposited by Dr Feng Zhang) (Ran et al., 2015) and the PM-FRB-Cerulean-T2A-FKBP-5-ptase plasmid, respectively, and assembled as a C-terminal FRB fusion gene and replacing the spCas9-mSA cassette of the PCS2+ Cas9-mSA plasmid using infusion cloning (Takara).

### Producing mRNAs and sgRNAs for microinjection

mRNAs and sgRNAs were produced using *in vitro* transcription following our published protocol (Gu et al., 2020a). Briefly, to produce the SpCas9-FKB and the SaCas9-FRB mRNA for microinjection, pCS2+ plasmids were linearized with NotI restriction digestion and used as a template for *in vitro* transcription using the mMESSAGE mMACHINE SP6 Transcription Kit (Thermo Fisher Scientific). The sgRNAs were produced by using the MEGAshortscript T7 Transcription Kit (Thermo Fisher Scientific). The RNeasy Mini Kit (QIAGEN) was used to purify all RNA products.

### Producing bridge donor

A bridge donor was designed consisting of the 136 bp upstream and 186 downstream sequences from the putative junction sequence for the tandem duplication. An EcoRI restriction site was placed between the upstream and downstream sequences. The sequence of the bridging donor (Table S8) was synthesized as a gBlock from Integrated DNA Technologies. PCR products were prepared with a pair of primers (Table S9) and purified for microinjection following our published protocols (Gu et al., 2020a).

### Mouse model generation

The *Mecp2 Dup* and *Mecp2 Del* mouse models were generated by microinjecting two-cell-stage embryos of the CD-1 mouse strain following our published protocols (Gu et al., 2020a,b). Microinjections were performed using a Leica microscope and micromanipulators (Leica Microsystem). The injection pressure was provided by a FemtoJet (Eppendorf) and negative capacitance was generated using a Cyto721 intracellular amplifier (World Precision Instruments). Microinjections were performed in M2 medium (Zenith Biotech). A mixture of SpCas9-FKB mRNA (100 ng/µl), SaCas9-FRB mRNA (100 ng/µl), *Irak1* sgRNA [50 ng/µl, protospacer sequence: 5ʹ-TAGCATCAATCAGCCCTAGT-3ʹ (SpCas9 sgRNA)], *Tex28* sgRNA [50 ng/µl, protospacer sequence: 5ʹ-CAGCTGTACTATGTTACCCAG-3ʹ (SaCas9 sgRNA)] and bridging donor (5 ng/µl) was microinjected in nuclease-free injection buffer (10 mM Tris-HCl, pH 7.4, 0.25 mM EDTA). Embryos were cultured in 5 nM rapamycin in KSOM (Zenith Biotech) for 6 h and then implanted into the oviducts of embryonic stage (E) 0.5 pseudo-pregnant females (30 embryos/female).

### Animal studies

All the animals used in this study were maintained in the specific pathogen-free facility at The Centre for Phenogenomics (TCP), Toronto, on a 12 h/12 h light/dark cycle and provided with food and water *ad libitum* in individually ventilated units (Techniplast). Only male mice on a CD-1 background (backcrossed for at least five generations) from 6 up to 52 weeks of age were used in this study. Mice were randomly assigned to either the experimental or control group. Experimenters masked to genotype carried out the behavioral study described in the manuscript. All procedures involving animals were performed in compliance with the Animals for Research Act of Ontario and the Guidelines of the Canadian Council on Animal Care. The Institutional Animal Care Committee reviewed and approved all procedures conducted on animals at TCP.

### Genomic DNA isolation, PCR and RT-PCR

Genomic DNA was isolated using the DNeasy blood and tissue kit (QIAGEN) according to the manufacturer's protocol. PCR was performed using DreamTaq polymerase (Thermo Fisher Scientific). The primers used for PCR amplification are reported in Table S9.

### Sanger sequencing

Amplified DNA was PCR purified using the QIAquick PCR Purification Kit (QIAGEN) according to the manufacturer's protocol and prepared for Sanger sequencing using the BigDye Terminator v3.1 Cycle Sequencing Kit (Thermo Fisher Scientific). Samples were sequenced on an Applied Biosystems SeqStudio Genetic Analyzer (Thermo Fisher Scientific) and electropherograms were analyzed using SnapGene software.

### RNA isolation, RT-PCR and quantitative PCR

RNA extraction was performed using TRIzol reagent (Thermo Fisher Scientific) following the manufacturer's protocol. Next, 1 µg of mRNA was reverse transcribed using the SuperScript III reverse transcriptase kit (Thermo Fisher Scientific). qPCR was performed using the Fast SYBR Green master mix (QIAGEN) on a StepOnePlus real-time PCR system (Applied Biosystems). ΔΔCt was analyzed to assess fold changes between gene expression in mutant and WT mice.

### Protein isolation and western blotting

Mouse tissue was homogenized in 400 μl of RIPA homogenizing buffer [50 mM Tris-HCl pH 7.4, 150 nM NaCl, 1 mM EDTA, supplemented with protease nhibitor cocktails (Roche)] and lysed with a MagNA Lyser (Roche). Subsequently, 400 μl of RIPA double-detergent buffer (2% deoxycholate, 2% NP40 and 2% Triton X-100 in RIPA homogenizing buffer) was added to the lysates, which were then incubated for 45 min at 4°C, and then centrifuged for 10 min at 18,000 ***g***. Protein concentration was measured using a BCA Assay (Thermo Fisher Scientific). Proteins were separated on a 4-12% Bis-Tris gel and transferred using an iBlot 2 transfer apparatus (Thermo Fisher Scientific). A 5% milk solution in Tris-buffered saline containing 0.1% Tween 20 was used for blocking for 1 h at room temperature. The membrane was then incubated with the appropriate primary antibodies – rabbit recombinant anti-MECP2 (Abcam, ab253197, 1:3000), rabbit monoclonal D51G7 anti-IRAK1 (Cell Signaling Technology, **#**4504, 1:1000), rabbit monoclonal anti-GAPDH (Santa Cruz Biotechnology, sc-47724, 1:10,000) – overnight at 4°C. The membranes were then incubated for 1 h at room temperature with horseradish peroxidase-conjugated goat anti-rabbit IgG (Abcam, ab6721, 1:10,000). Signal detection was achieved using SuperSignal West Femto Maximum-Sensitivity Substrate (Thermo Fisher Scientific) according to the manufacturer's protocol.

### Cryosectioning and immunofluorescence staining

Whole mouse brains were perfused with 4% paraformaldehyde, then cryopreserved in 20% sucrose in PBS, then in 30% sucrose in PBS, before cutting into 20 µm frozen sections using a HM525 cryostat (Thermo Fisher Scientific). Sections were fixed in ice-cold acetone for 5 min, then permeabilized with 0.1% Triton X-100 in PBS for 10 min, followed by blocking with 5% bovine serum albumin in 0.1% Triton X-100 in PBS for 1 h. The slides were incubated with anti-Arc (Proteintech, 16290-1-AP, 1:250) and anti-NeuN (Millipore, MAB377, 1:250) overnight at 4°C. On the next day, the primary antibody was replaced with Anti-Mouse IgG (H+L), Alexa Fluor 488 (Invitrogen, A11001, 1:500) and Anti-Rabbit IgG (H+L), Alexa Fluor 555 (Invitrogen, A21429, 1:500) for 2 h incubation at room temperature. The tissues were then stained with 300 nM DAPI (Invitrogen, D1306) and mounted on slides with ProLong Gold Antifade Mountant (Invitrogen, P36934), before imaging with a Quorum spinning disk confocal microscope. Quantification of the percentage of nuclear Arc was performed by manual counting across three areas per slide.

### ddPCR

Genomic DNA extracted from mouse tails was used to perform ddPCR by The Centre for Applied Genomics (TCAG) at the Hospital for Sick Children. Copy number estimation of *Tex28* and *Mecp2* were performed using the QX200 Droplet Digital PCR system (Bio-Rad Laboratories) using Taqman Copy Number assays: Mm00631912_cn and Mm00629942_cn (Life Technologies). Prior to the copy number experiment, 100 ng of genomic DNA was digested with 5 U of BtsCI in a 3 µl reaction (New England Biolabs) for 1 h at 50°C and with no enzyme denaturation. The 20 µl Copy Number reaction mix consisted of 10 µl of 2× ddPCR SuperMix for Probes (Bio-Rad Laboratories), 1 µl of the Copy Number target assay (labeled with FAM), 1 μl of the Copy Number Reference assay (mouse *Tfrc*, Life Technologies, part 4458366, labeled with VIC), 5 µl water and 3 µl of the digested genomic DNA. The Copy Number assay was validated by a temperature gradient to ensure optimal cluster separation of target and reference droplets. Cycling conditions for the reaction were 95°C for 10 min, followed by 45 cycles of 94°C for 30 s and 60°C for 1 min, 98°C for 10 min on a Life Technologies Veriti thermal cycler. Data were analyzed using QuantaSoft v1.4 (Bio-Rad Laboratories). WT controls and non-template controls were included with each run.

### WGS

DNA extracted from mouse tails was used for WGS, which was performed using the Illumina HiSeq X system (San Diego, CA, USA) by TCAG (Hospital for Sick Children). In brief, 400 ng of DNA sample was used for library preparation using the Illumina TruSeq PCR–free DNA Library Prep Kit, where DNA was sonicated into an average of 350-bp fragments. A-tailed and indexed TruSeq Illumina adapters were ligated to end-repaired sheared DNA fragments before the library was amplified. Libraries were analyzed using Bioanalyzer DNA High-Sensitivity chips (Agilent Technologies, Santa Clara, CA, USA) and quantified using qPCR. The libraries were loaded in equimolar quantities and pair-end sequenced on the Illumina HiSeq X platform to generate 150-bp reads. Integrative Genomics Viewer (IGV) version 2.8.2 was used for analysis with GRCm38/mm10 as the murine reference genome.

### Open-field test

For the open-field test, mice were placed in the frontal center of a transparent Plexiglas open field (41.25×41.25×31.25 cm) illuminated by 200 lux. The VersaMax Animal Activity Monitoring System recorded activity in the center and periphery of the open-field arena for 20 min per animal.

### Rotarod test

The mice were placed on a rotating rod (Panlab) that accelerated from 4 to 40 revolutions per minute. The duration of each trial was a maximum of 300 s for the *Mecp2 Dup* mice, and a maximum of 600 s for the *Mecp2 Del* mice. Mice were tested for three consecutive days, three trials each, with an interval of 15 min between trials to rest. The time that it took for each mouse to fall from the rod (latency to fall) was recorded.

### LTP measurements

We anesthetized 6-week-old mice with urethane (20% wt/vol, intraperitoneal injection). We prepared parasagittal hippocampal slices (300 μm) in ice-cold artificial cerebrospinal fluid (ACSF) (Sigma-Aldrich) and placed them in a holding chamber (30°C) for 40 min, then allowed it to passively cool down to room temperature (21 to 22°C for ≥30 min) before recording. We transferred a single slice to a recording chamber and perfused ACSF (Sigma-Aldrich) at 4 ml/min composed of 124 mM NaCl, 2.5 mM KCl, 1.25 mM NaH_2_PO_4_, 2 mM MgCl_2_, 11 mM D-glucose, 26 mM NaHCO_3_ and 2 mM CaCl_2_, saturated with 95% O2 (balanced with 5% CO_2_) at room temperature (pH 7.40, 305 mOsm). We evoked synaptic responses by stimulating Schaffer collateral afferents using bipolar tungsten electrodes located ∼50 μm from the pyramidal cell body layer in CA1. We recorded extracellular fEPSPs using ACSF-filled glass micropipettes placed in the stratum radiatum 60-80 μm from the cell body layer. Testing stimuli (0.1 ms in duration) were delivered at a frequency of 0.033 Hz to evoke a half-maximal fEPSP. In LTP experiments, TBS consisted of 16 bursts of four pulses at 100 Hz, delivered to Schaffer collateral afferents at an inter-burst interval of 200 ms. We amplified raw data using a MultiClamp 700B amplifier and a Digidata 1322A acquisition system sampled at 10 kHz, and analyzed the data with Clampfit 10.6 (Axon Instruments) and Sigmaplot 11 software (Grafiti LLC).

### Fear conditioning test

Individual pre-handled mice were placed in a conditioning chamber with controlled contextual cues and an electrified shock floor (Omnitech Electronics, Columbus, OH, USA). Each individual mouse was allowed to explore the conditioning chamber for 3 min before a conditioned stimulus, a 30-s tone (95 dB, 3600 Hz), was played for 30 s. During the last 2 s of the conditioned stimulus duration, an unconditioned stimulus (a mild foot shock of 0.75 mA) was delivered. Five training sessions were delivered in succession with an interval period of 30 s between the training sessions. After the last training session, the mouse was allowed to remain in the chamber for 30 s to assess the behavior of the mouse after the training. Fear responses were assessed by recording the freezing behaviors of the mouse using FreezeView2 (Coulbourn Instruments, Whitehall, PA, USA). Freezing was defined as lack of any locomotor activity except for slight head and tail movements (all within an index of motion of 10). Two and 24 h following the training, the fear memory of the mouse was tested using contextual and cued tests. During the contextual test, the contextual cues that were present during conditioning remained. The mouse was placed in the recording chamber for 4 min. During the cued test, the chamber and the contextual cues were changed. Following a 2 min acclimation, the same tone was played for 2 min. The movement of the mouse was tracked during both contextual and cued tests, and freezing responses were measured using FreezeView2. The duration of freezing responses of the mouse was measured and compared to the total duration of the test. Freezing responses were presented as a percentage.

### Influenza virus infection and sample collection

All work pertaining to mouse infection was carried out by The Centre for Phenogenomics (TCP, Montreal). *Mecp2 Dup* mice and WT littermates at 10 weeks of age (14 mice per genotype) were anesthetized via intraperitoneal injection of ketamine/xylazine, and subsequently infected intranasally with influenza H1N1 strain (100 plaque-forming units/35* *g mouse weight in PBS). The dose used was previously determined by TCP at LD_50_ for CD-1 WT mice. Mice were either monitored for 2 weeks (*Mecp2 Dup*, *n*=7; WT, *n*=7), including assessment of body weight and mortality, or euthanized on day 4 post infection for further assessment (*Mecp2 Dup*, *n*=7; WT, *n*=7). Baseline blood samples were collected via saphenous or facial vein puncture 1 day prior to infection (−1 dpi). On day 4 post infection (+4 dpi), mice were euthanized and blood samples were collected via cardiac puncture. BALF was obtained from euthanized mice by collecting 0.5 ml of PBS administered into the lungs via the trachea.

### Blood cells counts and differential

Cell count and differential from baseline blood samples was performed by TCP with the Hemavet Multispecies Hematology Systems HV950 (Drew Scientific). Blood cell count and differential measurements at day 4 post infection were performed by Biovet.

### Chemokine and cytokine analysis

BALF (undiluted) and serum samples (1:2 dilution in PBS) were collected and stored at −80°C until analysis. Samples were analyzed using the Mouse Multiplex Cytokine Array/Chemokine Array 44-Plex (MD44; Eve Technologies, Calgary, AB, USA).

### Splenocyte cytokine analysis in the absence or presence of IRAK1 inhibition

Spleens from 20-week-old mice (five per genotype) were collected and mechanically processed into single-cell suspension, followed by red blood cell lysis using eBioscience 10× RBC Lysis Buffer (Thermo Fisher Scientific, 00-4300-54). Splenic CD4T cells were subsequently isolated using the CD4T Cell Isolation Kit, Mouse (Miltenyi Biotec, 130-104-454). To stimulate IFNγ secretion, CD4T cells were cultured in RPMI (Gibco) (2×10^6^ cells/ml) and stimulated with anti-CD3 monoclonal antibody (17A2) (1 μg/ml, Thermo Fisher Scientific, 16-0032-82), anti-CD28 monoclonal antibody (37.51) (1 μg/ml, Thermo Fisher Scientific, 16-0281-82) and recombinant mouse IL-18 (100 ng/ml, R&D Systems, 9139-IL-010). Stimulation was performed in the absence or presence of a selective IRAK1 inhibitor (50 nM JH-X-119-01 hydrochloride, MedChemExpress, HY-103017). After 72 h, media were collected and sent for measurement of IFNγ levels by Luminex (Eve Technologies).

### Statistical analysis

All statistical analyses were performed using GraphPad Prism (GraphPad software). Data normality was tested using the Shapiro–Wilk test for *n*<8 datasets and D'Agostino–Pearson test for *n*>8 datasets. Parametric data were analyzed with two-tailed unpaired Student's *t*-tests, one-way ANOVA followed by Tukey's post hoc test, or two-way ANOVA repeated measures followed by Bonferroni multiple comparisons correction. Datasets with non-normal distribution were analyzed using two-tailed Mann–Whitney *U*-tests or Kruskal–Wallis test followed by Dunn's post hoc test. The exact numbers of animals used and the specific statistical analysis used are indicated in each figure legend. All behavioral analyses were performed with experimenters masked to genotype.

## Supplementary Material

10.1242/dmm.050528_sup1 Supplementary information
