# Supplementary material for: An Irak1-Mecp2 tandem duplication mouse model for the study of MECP2 duplication syndrome
Source: Dis Model Mech. 2024 Jul 23;17(7):dmm050528. doi: 10.1242/dmm.050528 (PMC11552499; doi:10.1242/dmm.050528)
Supplement: Supplementary information [file dmm-17-050528-s1.pdf]

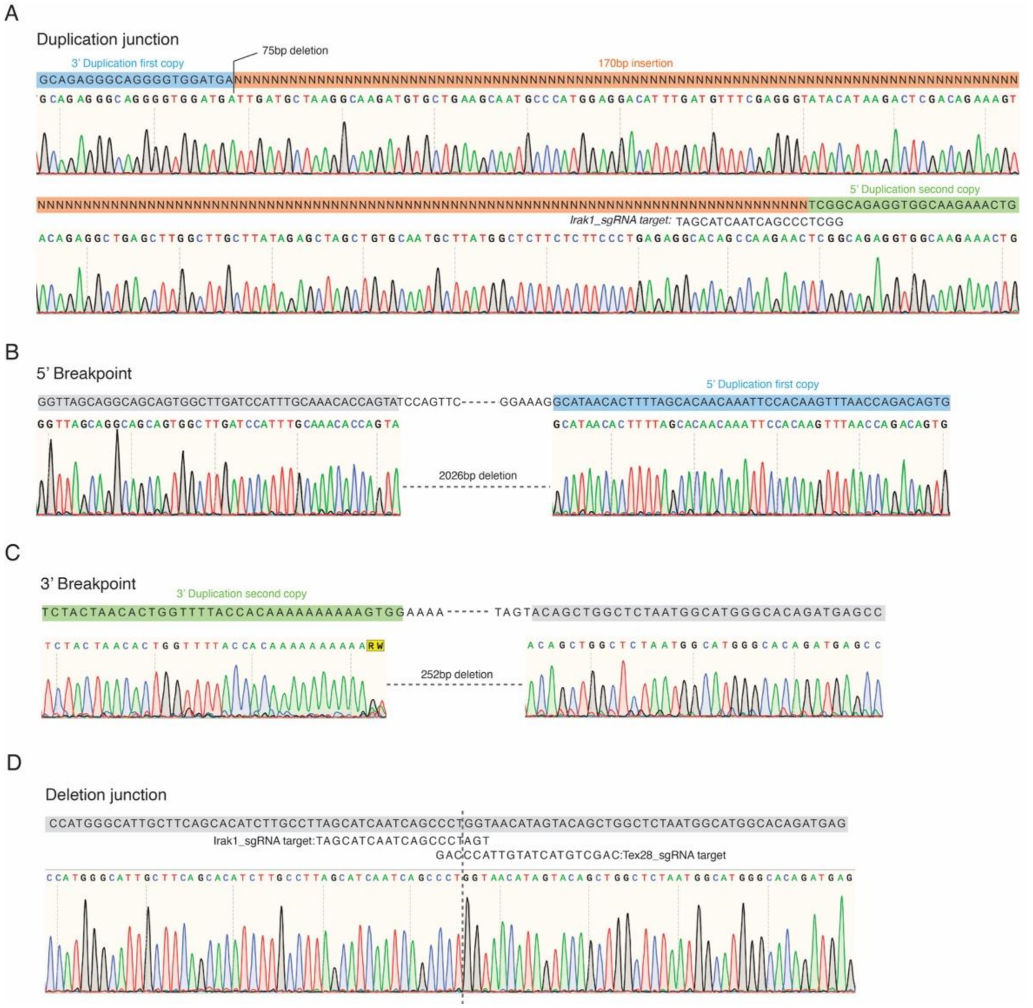

Fig. S1. Validation of the WGS results via Sanger sequencing. A) Sanger sequencing of the duplication junction confirms the insertion of 170 bp at the junction site in the *Mecp2 Dup* mouse model. Breakpoints' sequencing shows presence of **B)** a 2026 bp deletion at the 5' breakpoint, around the Irak1_sgRNA target site, and **C)** a 252 bp deletion at the 3' breakpoint, around the Tex28_sgRNA target site. **D)** The *Mecp2 Del* mouse model presents a precise deletion junction.

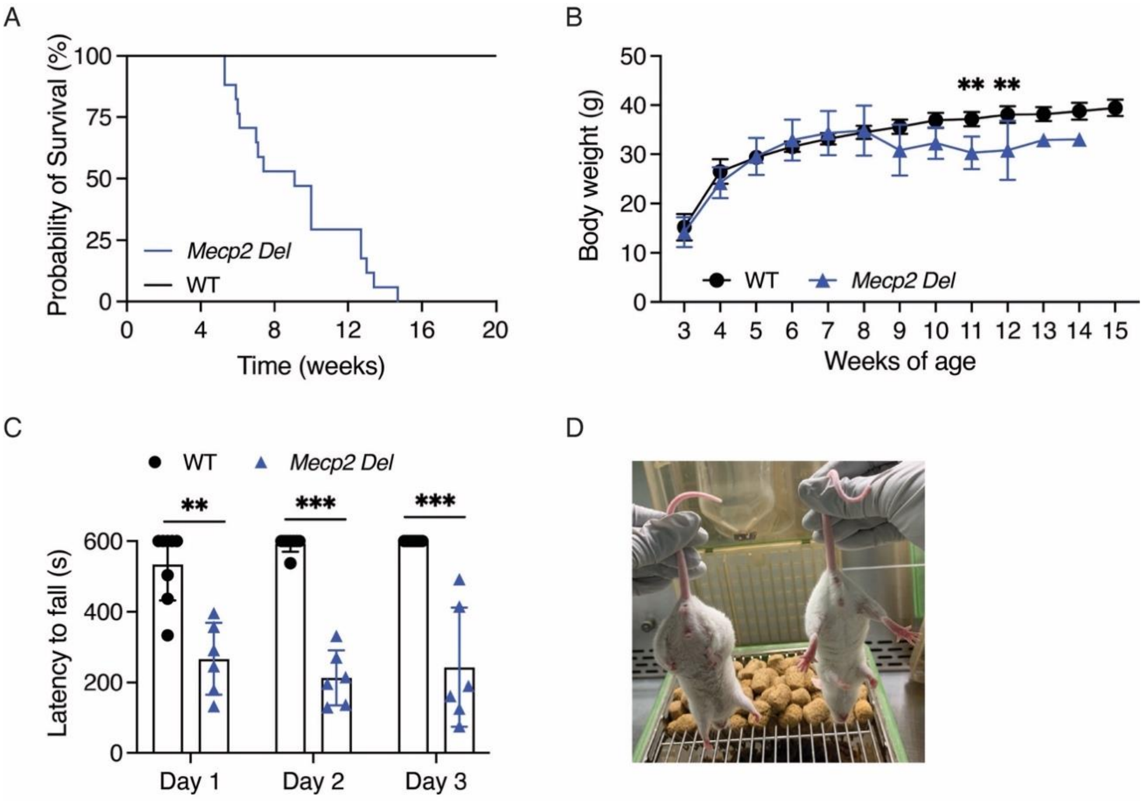

Fig. S2. The *Mecp2 Del* mouse model recapitulates RTT disease phenotypes. A) *Mecp2 Del* mice showed reduced survival compared to wild type littermates. *Mecp2 Dup*, n=17; WT, n=11. Survival curves were compared with the Mantel-Cox test. *Mecp Del* median survival age = 9.1 weeks; P <0.0001. **B)** Body weight curve of the mice shown in A. Statistical analysis was performed by mixed-effect two-way ANOVA repeated measures followed by Bonferroni's multiple comparison test. **C)** Three-day rotarod test showed reduced motor coordination in the *Mecp2 Del* mice. *Mecp2 Del*, n=6; WT, n=8. Data was analyzed by using the using two-tailed Mann–Whitney *U* test. ** P <0.1, *** P<0.001. **D)** *Mecp2 Del* mice (Left) show typical RTT clasping behaviour. A wild type littermate (right) is utilized for comparison. Data are represented as the mean +/- SD.

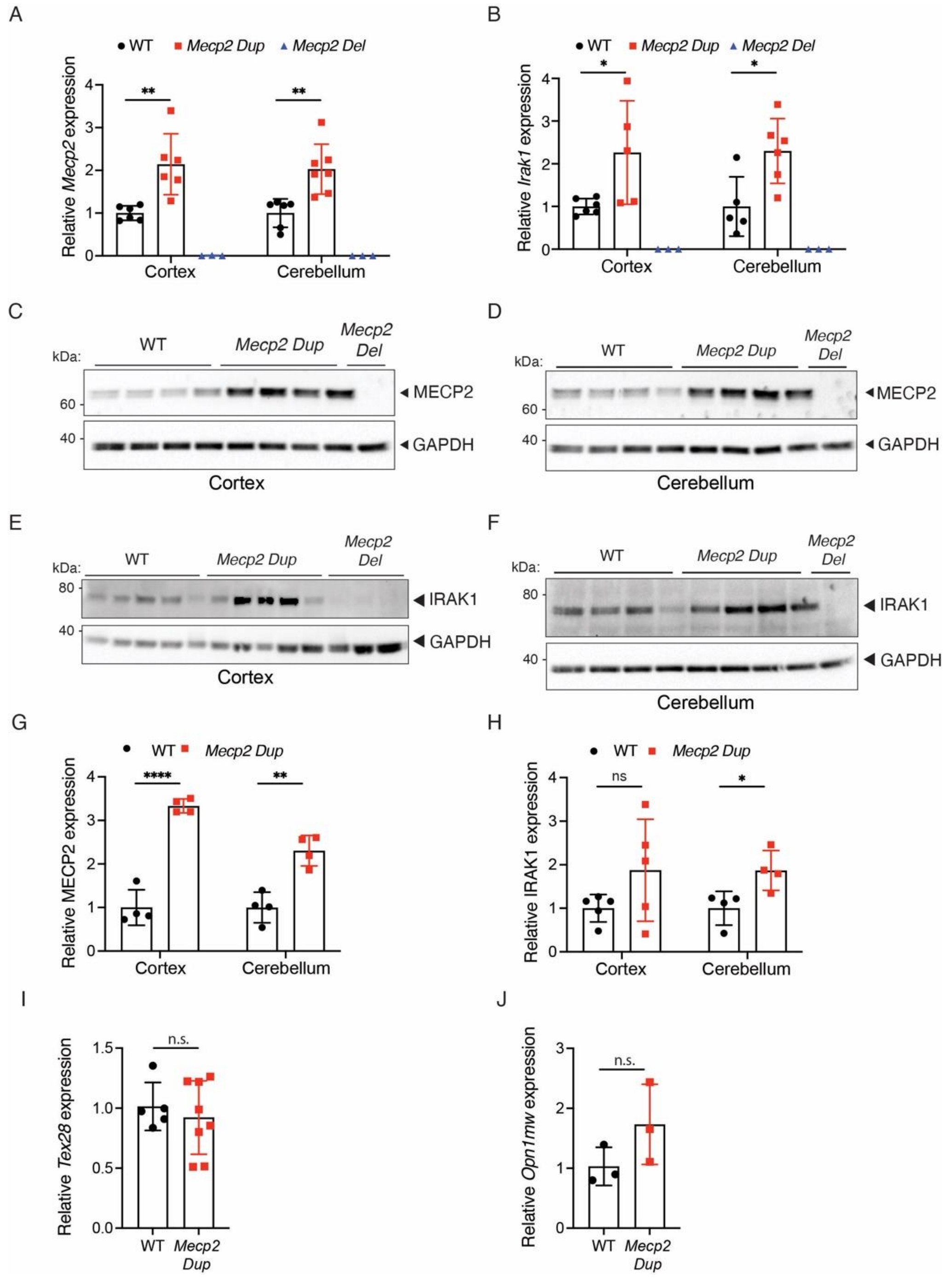

Fig. S3. *Mecp2* and *Irak1* are overexpressed in the cortex and cerebellum of *Mecp2 Dup* mice. A) The levels of *Mecp2* expression were analyzed via qPCR in the cortex and cerebellum of 10 weeks *Mecp2 Dup* mice, wild type littermates and *Mecp2 Del* mice. The data is normalized over *Gapdh* expression. WT, n=5-6; *Mecp2 Dup*, n=6-7; *Mecp2 Del*, n=3. **B)** *Irak1* transcript level was analyzed via qPCR. The data is normalized over *Gapdh* expression. WT,

Disease Models & Mechanisms • Supplementary information

n=5-6; *Mecp2 Dup*, n=5-6; *Mecp2 Del*, n=3. Western blot analysis confirmed increase of MECP2 expression **C)** in the cortex and **D)** in the cerebellum of *Mecp2 Dup* mice compared to wild type littermates. GAPDH serves as a loading control. IRAK1 expression was analyzed via Western Blot in **E)** cortex and **F)** cerebellum. GAPDH serves as a loading control. Densitometry analysis to quantify the amount of **G)** MECP2 and **H)** IRAK1 expression in various brain areas. WT, n=4-5; *Mecp2 Dup*, n=4-5. I) The levels of *Tex28* expression were analyzed via qPCR in the testis of *Mecp2 Dup* mice and wild type littermates. The data is normalized over *Gapdh* expression. WT, n=5; *Mecp2 Dup*, n=8. P=0.564. J) The levels of *Opn1mw* expression were analyzed via qPCR in the retina of *Mecp2 Dup* mice and wild type littermates. The data is normalized over *Rhodopsin* expression. WT, n=3; *Mecp2 Dup*, n=3. P=0.1760. All data are represented as the mean +/- SD. Statistical analyses were performed with Student's t-test. *P<0.5, **P<0.01, ****P<0.0001.

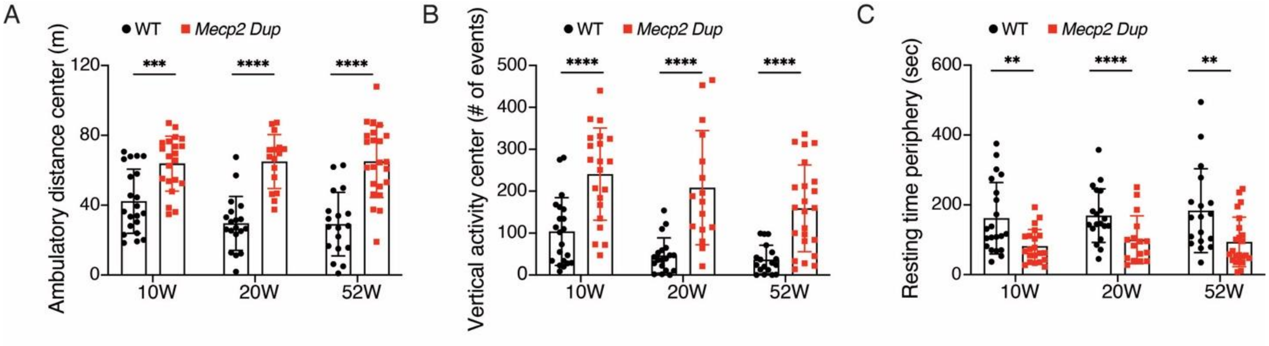

Fig. S4. The *Mecp2 Dup* mice show increased activity and decreased anxiety in the centre of the open field arena. The activity of *Mecp2 Dup* mice compared to wild type littermates was evaluated at 10, 20 and 52 weeks in the open field arena. All parameters analyzed in centre of the arena were highly dysregulated compared to wild type mice. **A)** Ambulatory distance in the center (10 weeks, P=0.0002; 20 weeks, P=1.039E-07; 52 weeks, P=9.6483E-07), **B)** Vertical activity in the center (10 weeks, P=0.000042; 20 weeks, P<0.0001; 52 weeks, P=2.2815E-05), and **C)** Resting time in the periphery (10 weeks, P=0.0023; 20 weeks, P=8.6863E-06; 52 weeks, P=0.0047) were measured. *Mecp2 Dup*, n=16-23; WT, n=18-21. Statistical analyses were performed with Student's t-test for data normally distributed or Mann–Whitney *U* test for data that do not follow a normal distribution. All data are represented as the mean +/- SD. **P<0.01, ***P<0.001, **** P<0.0001.

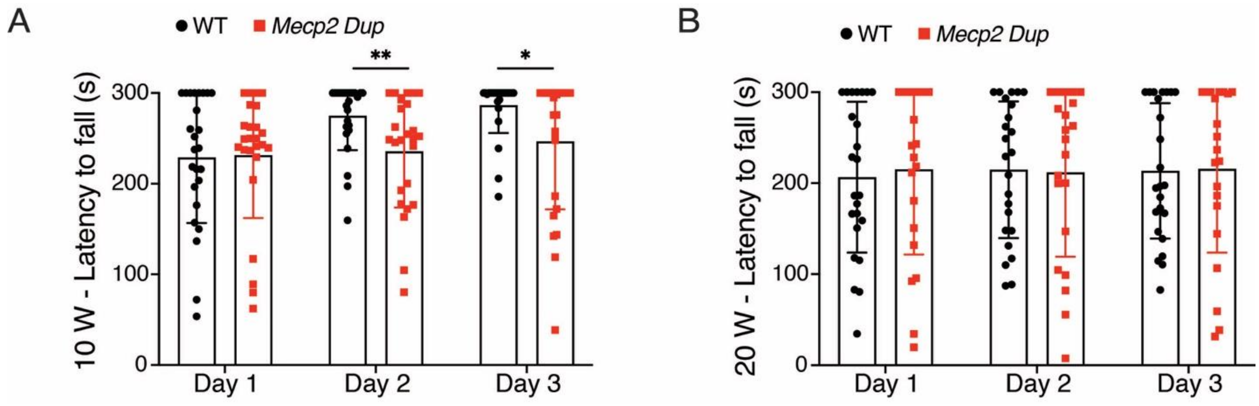

Fig. S5. The *Mecp2 Dup* mice show minimal motor coordination deficits in early disease stage. A 3 days rotarod test performed to evaluate motor coordination of the *Mecp2 Dup mice* at **A)** 10 weeks of age (day 1, P=0.9032, day 2, P=0.0057, day 3, P=0.0341) and **B)** 20 weeks of age (day 1, P=0.7459, day 2, P=0.9594, day 3, P=0.7755). Small differences were observed among the two groups. *Mecp2 Dup*, n=24-25; WT, n=21-25. Statistical analyses were performed with the Mann–Whitney *U* test or Student's t-test for data normally distributed. All data are represented as the mean +/- SD. *P<0.5, **P<0.01.

A

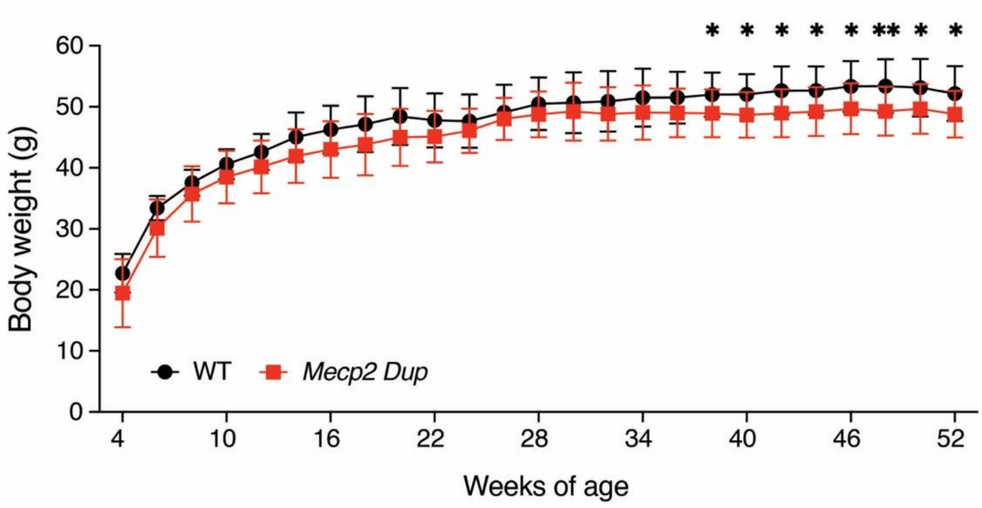

Fig. S6. Body weight analysis shows that *Mecp2 Dup* mice lose weight in late disease stages. A) Body weight was monitored weekly up from 4 to 52 weeks of age. *Mecp2 Dup*, n=11-17; WT, n=11-12. Statistical analysis was performed by mixed-effect two-way ANOVA repeated measures followed by Bonferroni's multiple comparison test. All data are represented as the mean +/- SD.

Disease Models & Mechanisms • Supplementary information

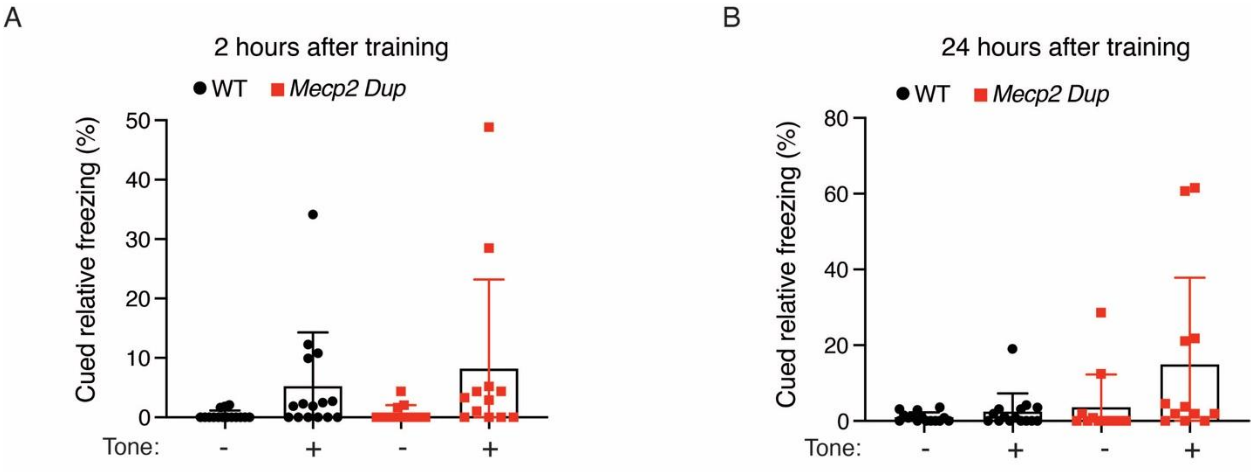

Fig. S7. The *Mecp2 Dup* mice display a trend toward increased freezing behaviour on cued test. A) No significant changes in percent freezing were shown when cued freezing behaviour was assessed both at 2 hours and **B)** 24 hours after training. *Mecp2 Dup*, n=12; WT, n=15. Statistical analysis performed by Kruskal Wallis test followed by Dunn's multiple comparison test. All data are represented as the mean +/- SD.

Disease Models & Mechanisms • Supplementary information

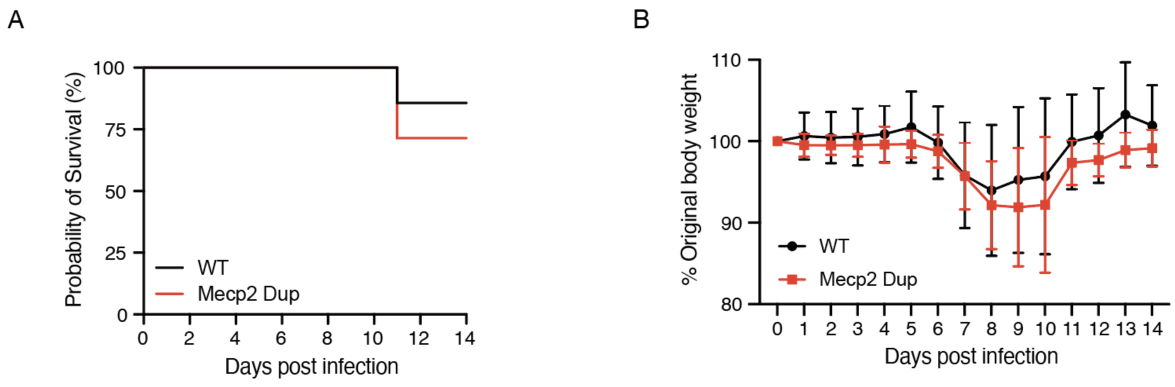

Fig. S8. Body weight and survival after influenza infection show no difference between *Mecp2 Dup* and wild type mice. A) Survival of 10 weeks old *Mecp2 Dup* and wild type littermates infected with a dose of influenza shows no differences up to day 14 post infection. *Mecp2 Dup*, n=7; wild type, n=7. Statistical analysis performed with Mantel-Cox test (P = 0.5302). **B)** Weight loss upon infection in *Mecp2 Dup* and wild type littermates was measured up to day 14 post infection as percentage of body weight compared to day 0. *Mecp2 Dup*, n=5-7; wild type, n=6-7. Statistical analysis was performed by mixed-effect two-way ANOVA repeated measures followed by Bonferroni's multiple comparison test. Data are represented as the mean +/- SD.

Disease Models & Mechanisms • Supplementary information

Table S1. ***Mecp2 Dup*** **– WGS structural variants**

Chr	Pos start	Pos end	SV length (bp)	SV type	What
X	74,012,223	74,014,249	-2026	DEL	5' breakpoint deletion
X	74,014,249	74,172,116	157,876	DUP	*Irak1-Tex28* dup
X	74,012,505	74,012,335	170	INS	Insertion at the duplication junction
X	74,171,949	74,172,201	-252	DEL	3' breakpoint deletion

Table S2. ***Mecp2 Dup*** **mouse model generation –Irak1 sgRNA off-targets**

Target	sgRNA sequence	PAM	Gene	Chr	# Mismatch	SVs
Irak1 sgRNA	TAGCATCAATCAGCCCTAGT	CGG	None	X	0	Yes
OT1	AAGCATGAAGCAGCCCTAGT	GGG	ENSMUSG00000024109	17	3	None
OT2	CAGTTTCAAGCAGCCCTAGT	TGG	None	1	4	None
OT3	AAGCATAAATCTGCCCTAGT	TAG	None	2	3	None
OT4	TGACATCATCCAGCCCTAGT	AGG	ENSMUSG00000030317	6	4	None
OT5	TGGGACCAAGCAGCCCTAGT	GAG	ENSMUSG00000022883	16	4	None
OT6	TACCCACCATCAGCCCTAGT	AAG	None	14	4	None
OT7	TTTCATCTATCTGCCCTAGT	GAG	ENSMUSG00000085931	4	4	None
OT8	TAGCTCCAATCAGCCCTACT	AAG	ENSMUSG00000027971	3	3	None
OT9	TGGCTTCAAACAGCCCAAGT	GAG	ENSMUSG00000110967	10	4	None
OT10	AAGGATCAAGCAACCCTAGT	CAG	None	9	4	None

Table S3. *Mecp2 Dup* mouse model generation –Tex28 sgRNA off-targets

Target	sgRNA sequence	PAM	Gene	Chr	# Mismatch	SVs
Tex28 sgRNA	CAGCTGTACTATGTTACCCAG	ATGAGT	None	X	0	Yes
OT1	AGCTTTACTAAGTTACCTAG	CAGAG	ENSMUSG00000079109	5	3	None
OT2	ACCTGAACTATGTCACCCAG	CAGAA	None	4	3	None
OT3	AGCTGTACTCTGTGGCCCAG	GTGGG	ENSMUSG00000026956	2	3	None
OT4	ATATATACTATGTTACCCAG	GTGGG	None	9	3	None
OT5	TGGTGTACTATGTTAGCTAG	TGGAG	ENSMUSG00000032295	9	4	None
OT6	TGTTGTACTATTTTACCCTG	GGGAA	ENSMUSG00000062542	7	4	None
OT7	AACTGTATTATGTTACACAC	ATGAA	None	8	4	None
OT8	GGCTGTCCTATGTCACCCTG	CAGAG	ENSMUSG00000031503	8	4	None
OT9	GGCTGAACTATGTTCCCTAG	CAGGG	None	14	4	None
OT10	ACATGTACTATGTGACCAAG	AAGAG	None	10	4	None

Disease Models & Mechanisms • Supplementary information

Table S4. Cytokine/Chemokine 44-Plex Discovery Assay® Array of Serum of *Mecp2 Dup* and wild type mice the day prior (d.p.i -1) to *Influenza* infection. Statistical analysis performed with Student's t-test.

Chemokines and cytokines analysis, Serum d.p.i. -1

Molecule	Concentration (pg/mL +/- SEM)		Fold Change	p value
	Wild type	*Mecp2 Dup*		
Eotaxin	574.56 ± 71.49	530.48 ± 57.85	0.92	0.6404
G-CSF	280.67 ± 54.98	422.94 ± 85.33	1.51	0.1864
GM-CSF	14.64 ± 2.64	11.65 ± 4.33	0.80	0.5453
IFNγ	157.61 ± 154.7	39.89 ± 20.56	0.25	0.4652
IL-1α	526.09 ± 58.09	431.11 ± 45.85	0.82	0.2236
IL-1β	3.75 ± 0.85	5.25 ± 1.06	1.40	0.2926
IL-2	4.72 ± 0.45	4.05 ± 0.55	0.86	0.3674
IL-3	2.39 ± 0.8	1.75 ± 0.64	0.73	0.5592
IL-4	0.14 ± 0.06	0.26 ± 0.05	1.88	0.1972
IL-5	3.59 ± 0.67	9.92 ± 5.55	2.76	0.2804
IL-6	5.73 ± 2.52	8.17 ± 1.63	1.43	0.4326
IL-7	2.78 ± 0.5	2.17 ± 0.52	0.78	0.4148
IL-9	22.86 ± 2.45	20.56 ± 2.37	0.90	0.5140
IL-10	10.94 ± 1.08	8.85 ± 1.02	0.81	0.1850
IL-12p40	17.85 ± 3.48	9.79 ± 1.82	0.55	0.0632
IL-12p70	17.71 ± 4.25	16.05 ± 4.33	0.91	0.7914
IL-13	100.01 ± 32.9	63.93 ± 4.12	0.64	0.2980
IL-15	79.48 ± 11.44	56.91 ± 11.8	0.72	0.1950
IL-17	2.45 ± 0.36	2.04 ± 0.26	0.83	0.3827
CXCL10	74.47 ± 9.8	74.55 ± 8.47	1.00	0.9953
CXCL1	55.23 ± 9.39	55.21 ± 2.94	1.00	0.9984
LIF	0.34 ± 0	Not detected	N/A	N/A
CXCL5	3225.31 ± 279.55	3110.24 ± 240.17	0.96	0.7602
CCL2	25.78 ± 2.84	26.96 ± 1.8	1.05	0.7338
M-CSF	20.07 ± 4.92	139.09 ± 100.7	6.93	0.2607
CXCL9	1005.04 ± 338.93	1262.18 ± 141.78	1.26	0.4973
CCL3	59.49 ± 7.48	54.54 ± 10.32	0.92	0.7048
CCL4	109.57 ± 11.76	106.91 ± 6.59	0.98	0.8473
CXCL2	709.09 ± 1.33	707.98 ± 1.21	1.00	0.5511
CCL5	35.31 ± 3.05	43.53 ± 2.84	1.23	0.0725
TNFα	14 ± 0.75	13.53 ± 0.59	0.97	0.6295
VEGF	1.65 ± 0.27	1.41 ± 0.08	0.85	0.4096

Disease Models & Mechanisms • Supplementary information

CCL21	17540.11 ± 659.84	17145.67 ± 930.58	0.98	0.7355
EPO	1011.68 ± 212.34	678.58 ± 82.56	0.67	0.1694
CX3CL1	616.49 ± 53.76	455.24 ± 25.97	0.74	0.0193
IFNβ-1	1703.83 ± 388.09	1680.41 ± 672.22	0.99	0.9764
IL-11	44.73 ± 9.71	30.45 ± 2.21	0.68	0.1771
IL-16	5874.93 ± 949.12	5103.9 ± 723.72	0.87	0.5304
IL-20	1801.93 ± 297.37	2090.13 ± 474.65	1.16	0.6162
CCL12	337.97 ± 77.18	440.08 ± 70.03	1.30	0.3466
CCL22	514.89 ± 136.58	566.6 ± 84.56	1.10	0.7531
CCL20	56.07 ± 6.69	52.29 ± 8.39	0.93	0.7313
CCL19	808.81 ± 92.72	447.21 ± 89.67	0.55	0.0159
CCL17	116.51 ± 25.59	104.08 ± 25.08	0.89	0.7348
TIMP-1	5405.51 ± 577.66	6651.72 ± 516.47	1.23	0.1338

Disease Models & Mechanisms • Supplementary information

Table S5. Cytokine/Chemokine 44-Plex Discovery Assay® Array of Serum of *Mecp2 Dup* and wild type mice at day 4 post *Influenza* infection (d.p.i +4). Statistical analysis performed with Student's t-test.

Chemokines and cytokines analysis, Serum d.p.i. +4

**Molecule**	**Concentration (pg/mL +/- SEM)**		**Fold Change**	**p value**
	**Wild type**	***Mecp2 Dup***		
Eotaxin	1803.42 ± 137.49	1801.73 ± 166.79	0.9991	0.9939
G-CSF	597.17 ± 155.25	628.73 ± 80.66	1.0529	0.8599
GM-CSF	10.57 ± 1.42	19.94 ± 4.28	1.8860	0.0303
IFNγ	51.01 ± 20.39	113.69 ± 55.27	2.2288	0.3083
IL-1α	310.14 ± 29.26	319.15 ± 53.69	1.0290	0.8853
IL-1β	0.91 ± 0.18	1.4 ± 0.73	1.5245	0.4135
IL-2	4.65 ± 0.43	3.78 ± 0.64	0.8125	0.2840
IL-3	1.69 ± 0.73	4.31 ± 1.78	2.5499	0.1774
IL-4	0.22 ± 0.15	0.22 ± 0.1	1.0000	1.0000
IL-5	7.28 ± 2.64	3.59 ± 0.52	0.4940	0.1964
IL-6	103.27 ± 37.84	118.4 ± 28.65	1.1465	0.7554
IL-7	2.06 ± 0.74	2.84 ± 0.73	1.3767	0.4895
IL-9	21.72 ± 2.28	24.04 ± 1.27	1.1068	0.3930
IL-10	7.67 ± 0.78	8.67 ± 0.85	1.1302	0.4061
IL-12p40	12.83 ± 2.01	16.21 ± 2.91	1.2632	0.3586
IL-12p70	17.44 ± 7.16	29.25 ± 13.27	1.6768	0.4803
IL-13	136.7 ± 35.95	129.92 ± 10.54	0.9504	0.8594
IL-15	82.48 ± 12.29	78.63 ± 15.61	0.9533	0.8496
IL-17	1.56 ± 0.34	1.74 ± 0.55	1.1160	0.7859
CXCL10	428.84 ± 47.29	406.07 ± 42.03	0.9469	0.7252
CXCL1	93.13 ± 9.93	115.82 ± 18.95	1.2436	0.3099
LIF	1.01 ± 0	0.73 ± 0	0.7228	N/A
CXCL5	4302.98 ± 347.89	5165.44 ± 631.54	1.2004	0.2547
CCL2	55.77 ± 9.19	65.39 ± 14.27	1.1724	0.5816
M-CSF	20.06 ± 4.88	36.2 ± 13.43	1.8046	0.2810
CXCL9	2119.18 ± 266.85	3523.4 ± 330.05	1.6626	0.0062
CCL3	136.99 ± 8.58	108.38 ± 14.55	0.7912	0.1162
CCL4	165.12 ± 9.44	170.38 ± 11.23	1.0318	0.7264
CXCL2	707.25 ± 0.83	707.62 ± 0.99	1.0005	0.7811
CCL5	39.73 ± 1.24	56.05 ± 4.07	1.4109	0.0024
TNFα	18.94 ± 1.02	20.26 ± 1.78	1.0698	0.5322
VEGF	1.74 ± 0.22	1.91 ± 0.12	1.0982	0.5264

Disease Models & Mechanisms • Supplementary information

CCL21	22250.88 ± 343.44	20570.85 ± 831.44	0.9245	0.0864
EPO	924.65 ± 186.86	722.85 ± 105.92	0.7818	0.3660
CX3CL1	907.75 ± 87.78	795.34 ± 146.35	0.8762	0.5225
IFNβ-1	1960.96 ± 809.53	2560.38 ± 1003.13	1.3057	0.6502
IL-11	84 ± 19.59	50.27 ± 6.81	0.5984	0.1299
IL-16	9859.11 ± 625.11	7511.15 ± 725.21	0.7618	0.0305
IL-20	1482.15 ± 154.04	2290.45 ± 333.31	1.5454	0.0480
CCL12	1446.66 ± 145.53	1535.04 ± 217.89	1.0611	0.7417
CCL22	1372.61 ± 332.07	1518.94 ± 174.7	1.1066	0.7034
CCL20	222.74 ± 51.15	203.44 ± 55.1	0.9133	0.8017
CCL19	906.41 ± 183.14	940.89 ± 135.61	1.0380	0.8823
CCL17	369.97 ± 61.43	320.12 ± 35.28	0.8653	0.4951
TIMP-1	15313.99 ± 638.45	14257.63 ± 506.22	0.9310	0.2192

Disease Models & Mechanisms • Supplementary information

Table S6. Cytokine/Chemokine 44-Plex Discovery Assay® Array of BALF of *Mecp2 Dup* and wild type mice at day 4 post *Influenza* infection (d.p.i +4). Statistical analysis performed with Student's t-test.

Chemokines and cytokines analysis, BALF d.p.i. +4

**Molecule**	**Concentration (pg/mL +/- SEM)**		**Fold Change**	**p value**
	**Wild type**	***Mecp2 Dup***		
Eotaxin	150.65 ± 10.22	119.88 ± 4.1	0.80	0.0162
G-CSF	38.11 ± 6.08	55.03 ± 8.58	1.44	0.1339
GM-CSF	3.47 ± 0.95	4.85 ± 0.83	1.40	0.3045
IFNγ	352.07 ± 196.13	2204.69 ± 737.62	6.26	0.0319
IL-1α	3.2 ± 0.59	11.48 ± 3.26	3.59	0.0951
IL-1β	0.55 ± 0.19	3.24 ± 1.41	5.90	0.1667
IL-2	0.53 ± 0.08	0.89 ± 0.06	1.69	0.0062
IL-3	0.78 ± 0.05	0.84 ± 0.05	1.08	0.4309
IL-4	0.05 ± 0	0.08 ± 0	1.57	0.0305
IL-5	0.49 ± 0.11	0.6 ± 0.04	1.22	0.4006
IL-6	253.66 ± 65.72	242.48 ± 57.93	0.96	0.9006
IL-7	0.95 ± 0.22	1.17 ± 0.18	1.23	0.4648
IL-9	3.51 ± 0.45	4.41 ± 0.9	1.25	0.3940
IL-10	0.28 ± 0.07	0.94 ± 0.6	3.31	0.1945
IL-12p40	Not detected	Not detected	N/A	N/A
IL-12p70	1.44 ± 0.35	1.78 ± 0.4	1.24	0.5473
IL-13	0.8 ± 0.12	1.94 ± 0.47	2.43	0.0427
IL-15	2.31 ± 0.81	3.09 ± 0.75	1.34	0.5033
IL-17	0.15 ± 0.02	0.36 ± 0.08	2.33	0.0551
CXCL10	515.73 ± 96.37	991.88 ± 194.33	1.92	0.0486
CXCL1	93.62 ± 17.76	114.84 ± 10.4	1.23	0.3231
LIF	6.24 ± 1.83	5.86 ± 0.85	0.94	0.8564
CXCL5	Not detected	Not detected	N/A	N/A
CCL2	95.2 ± 18.5	77.81 ± 6.49	0.82	0.3929
M-CSF	1.32 ± 0.23	1.95 ± 0.1	1.47	0.0331
CXCL9	72.41 ± 16.8	230.72 ± 47.81	3.19	0.0088
CCL3	93.9 ± 16.19	112 ± 7.19	1.19	0.3272
CCL4	134.58 ± 22.71	145.71 ± 16.74	1.08	0.7004
CXCL2	14.98 ± 2.29	27.47 ± 2.26	1.83	0.0022
CCL5	1.91 ± 0.41	3.29 ± 0.68	1.72	0.1109
TNFα	3.75 ± 0.68	8.19 ± 1.65	2.19	0.0291
VEGF	4.44 ± 1.37	6.43 ± 1.51	1.45	0.3494

Disease Models & Mechanisms • Supplementary information

CCL21	8255.63 ± 3702.34	6379.5 ± 2506.59	0.77	0.6715
EPO	Not detected	Not detected	N/A	N/A
CX3CL1	17.15 ± 2.44	20.7 ± 1.97	1.21	0.2808
IFNβ-1	60.27 ± 5.32	74.43 ± 6.65	1.23	0.1226
IL-11	0 ± 0	0 ± 0	N/A	N/A
IL-16	253.77 ± 37.57	273.49 ± 15.13	1.08	0.6353
IL-20	27.83 ± 2.18	51.78 ± 2.48	1.86	0.0006
CCL12	231 ± 27.72	252.59 ± 14.07	1.09	0.5007
CCL22	46.05 ± 7.14	63.24 ± 11.69	1.37	0.2336
CCL20	2.48 ± 0.97	3.28 ± 0.94	1.32	0.5660
CCL19	34.89 ± 3.71	55.75 ± 3.58	1.60	0.0016
CCL17	12.47 ± 1.74	11.83 ± 1.48	0.95	0.7830
TIMP-1	2822.88 ± 493.37	1903.41 ± 68.44	0.67	0.0897

Table S7. Cytokine analysis of high sensitivity T cell panel array of T cells CD4+ supernatant in presence or absence of Irak1 inhibitor.

	Concentration (pg/mL +/- SEM)			
**Molecule**	**Wild type**	**Wild type + Irak1 inhibitor**	***Mecp2 Dup***	***Mecp2 Dup* + Irak1 inhibitor**
GM-CSF	2068.64 ± 139.23	1367.88 ± 276.22	2137.66 ± 69.33	1500.88 ± 249.65
IFNγ	16392.26 ± 2973.78	5302.09 ± 2283.40	77345.15 ± 39356.03	4538.49 ± 1632.08
IL-1α	237.35 ± 14.95	99.47 ± 17.16	235.16 ± 7.47	94.96 ± 21.79
IL-1β	25.48 ± 10.79	6.24 ± 1.20	32.80 ± 9.00	13.83 ± 3.83
IL-2	OOR >	OOR >	OOR >	OOR >
IL-4	156.64 ± 23.28	94.32 ± 39.40	335.91 ± 187.97	97.83 ± 40.88
IL-5	309.50 ± 56.67	7.23 ± 1.55	374.40 ± 45.17	11.73 ± 2.89
IL-6	915.89 ± 323.86	131.84 ± 46.19	1330.51 ± 449.77	305.94 ± 111.53
IL-7	1.17 ± 0.21	0.79 ± 0.23	1.21 ± 0.16	0.80 ± 0.12
IL-10	293.95 ± 38.23	54.46 ± 14.37	356.74 ± 103.57	86.57 ± 28.89
IL-12p70	8.99 ± 1.09	1.51 ± 0.38	9.03 ± 1.17	3.18 ± 0.71
IL-13	11.89 ± 4.07	N/A	26.05 ± 11.37	N/A
IL-17A	2975.81 ± 321.12	437.62 ± 411.40	5498.65 ± 899.95	2158.42 ±742.57
CXCL1	38.76 ± 13.49	9.81 ± 2.06	51.91 ± 12.40	14.82 ± 3.71
CXCL5	66.81 ± 3.97	35.66 ± 8.89	79.13 ±14.12	39.51 ± 10.14
CCL2	59.54 ± 3.63	16.76 ± 2.88	116.33 ± 34.74	44.26 ± 12.09
CXCL2	250.00 ± 96.29	100.64 ± 20.39	257.94 ± 42.50	155.78 ± 10.54
TNFα	497.58 ± 51.90	137.29 ± 34.70	478.65 ± 53.24	167.95 ± 43.43

Disease Models & Mechanisms • Supplementary information

Table S8. sgRNAs and bridging donor utilized to generate the *Mecp2 Dup* mice

Sequence name	Sequence
Bridging donor	GTTGCTCCCACCTGAACAGCAAAGTAGAGTTGGTCTTGGTTGC AGAGGGCAGGGGTGGATGAAGGTGAGTCAGCTCTGAGAGTTA GAGAGTGGGAAAGCTGGCCCAGCCCCTTGCTTGCTGCAGGAA ACTCATCTGgaattcTAGTCGGCAGAGGTGGCAAGAAACTGAAG TAAGGGCTGGTGAGATGGCTCAGCGGTTAAAGAGCGCCGACT GCTCTTCCAAAGGTCATGAGTTCAAATCCCAGCAACCACATG GTGGCTCATAACCATCTGTAATGAAATCTGATGCCCTCTTCTG GTGTGTCTGAAGACAGCTACAGTGTACTTACAT
Irak1_sgRNA	TAGCATCAATCAGCCCTAGT
Tex28_sgRNA	CAGCTGTACTATGTTACCCAG

Table S9. Oligonucleotides utilized in this study

Primer name	Sequence	Purpose
mMecp2 Dup 5' del F	AACTCACCAGAGGTAGTGGAAAAG	5’ breakpoint PCR + sequencing
mMecp2 Dup 5' del R	CTATAGCCCAGGCTTACAGTGATT	
mMecp2 geno F	ATGGTTGGAGCCTGGGTATT	Dup junction PCR + sequencing
mMecp2 geno R	CCATCTCACCAGCCCTTACT	
mMecp2 3’ break	ATGGTGAGATGACCAATGACTTC	3’ breakpoint PCR + sequencing
mMecp2 Dup 3' del R	CCTGGAGATCTTGTAGTTTTGGAT	
mMecp2 Del F	GAGTAACTCCTGTCTGTGTTGTCT GG	Del junction PCR + sequencing
mMecp2 Del R	CTTACATTGCAGAGTTGAGCTGCGTT	
mMecp2 e1 F	AGG AGA GAC TGG AGG AAA AGT C	RT-PCR *Mecp2* isoforms
mMecp2 e2 F	CTTAAACTTCAGTGGCTTGTCTCTG	
mMecp2 e1-e2 R	CTCACCAGTTCCTGCTTTGATGT	
mMecp2 qPCR ex3 F2	CGATCTGCTGGAAAGTATGATGT	qPCR
mMecp2 qPCR ex4 R2	CTTCTTAGGTGGTTTCTGCTCTCT	
mIrak1 qPCR F	TGTGAAGAGACTGAAGGAGGAAG	
mIrak1 qPCR R	CCGCAAAGTCTACGATATTTGG	
mGapdh F	TGTTTGTGATGGGTGTGAACC	
mGapdh R	ACTGTGGTCATGAGCCCTTC	
mCacna1g qPCR F	GACCATGTGGTCCTCGTCATCA	
mCacna1g qPCR R	TTTCAGCCAGGAAGACTGCCGT	
mGad2 qPCR F	CATTGATAAGTGTTTGGAGCTAGCA	
mGad2 qPCR R	GTGCGCAAACTAGGAGGTACAA	
mFxyd7 qPCR F	AAGGCGGATTCCAGGTCTGA	
mFxyd7 qPCR R	GGAGGGCAGTTCCGACTTAC	
mRhodopsin qPCR F	GGCTTCCCTATGCCAGTGT	
mRhodopsin qPCR R	TCATCTCCCAGTGGATTCTTG	
mTex28 qPCR F	AGTCACTCCAGGAAAAGAAAACC	
mTex28 qPCR R	ATCTCATCCAGGTATCTCTGCAA	
mOpn1mw qPCR F	TTATATGATGGTCCTCATGGTCA	
mOpn1mw qPCR R	TCAGATTCTTTCTGTTGCTTTGC	

Disease Models & Mechanisms • Supplementary information
